# An explanatory model of temperature influence on flowering through whole-plant accumulation of *FLOWERING LOCUS T* in *Arabidopsis thaliana*

**DOI:** 10.1093/insilicoplants/diz006

**Published:** 2019-05-15

**Authors:** Hannah A. Kinmonth-Schultz, Melissa J. S. MacEwen, Daniel D. Seaton, Andrew J. Millar, Takato Imaizumi, Soo-Hyung Kim

**Affiliations:** 1 Department of Biology, University of Washington, Seattle, WA 98195, USA; 2 SynthSys and School of Biological Sciences, University of Edinburgh, Edinburgh EH9 3JY, UK; 3 School of Environmental and Forest Sciences, University of Washington, Seattle, WA 98195, USA; 4 Present address: Department of Ecology and Evolutionary Biology, University of Kansas, Lawrence, KS 66045, USA; 5 Present address: Department of Pharmacology, University of Washington, Seattle, WA 98195, USA; 6 Present address: European Bioinformatics Institute, European Molecular Biology Laboratory, Cambridge CB10 1SD, UK

**Keywords:** *Arabidopsis*, *Arabidopsis thaliana*, crop simulation model, flowering time, Framework Model, *FT*, mathematical model, phenology, photoperiodic flowering, thermal time

## Abstract

We assessed mechanistic temperature influence on flowering by incorporating temperature-responsive flowering mechanisms across developmental age into an existing model. Temperature influences the leaf production rate as well as expression of *FLOWERING LOCUS T* (*FT*), a photoperiodic flowering regulator that is expressed in leaves. The *Arabidopsis* Framework Model incorporated temperature influence on leaf growth but ignored the consequences of leaf growth on and direct temperature influence of *FT* expression. We measured *FT* production in differently aged leaves and modified the model, adding mechanistic temperature influence on *FT* transcription, and causing whole-plant *FT* to accumulate with leaf growth. Our simulations suggest that in long days, the developmental stage (leaf number) at which the reproductive transition occurs is influenced by day length and temperature through *FT*, while temperature influences the rate of leaf production and the time (in days) the transition occurs. Further, we demonstrate that *FT* is mainly produced in the first 10 leaves in the Columbia (Col-0) accession, and that *FT* accumulation alone cannot explain flowering in conditions in which flowering is delayed. Our simulations supported our hypotheses that: (i) temperature regulation of *FT*, accumulated with leaf growth, is a component of thermal time, and (ii) incorporating mechanistic temperature regulation of *FT* can improve model predictions when temperatures change over time.

## Introduction

Ambient temperature during the growing season correlates with the timing of plants’ transition from vegetative to reproductive growth. Germination, organ emergence, leaf expansion, photosynthesis and respiration display similar relationships (Parent *et al*. 2010). These findings have led to the concept of ‘thermal time’ (Lehenbauer 1914), a metric that asserts that temperature-driven metabolic rates govern development (Zavalloni *et al*. 2006), and to models such as growing degree days (GDD) that use the empirical relationship between temperature and development to predict plant response (e.g. Ritchie and Otter 1985; Jamieson *et al*. 1998b; Chuine 2000; Jones *et al*. 2003; Wilczek *et al*. 2009; He *et al*. 2012; Kumudini *et al*. 2014).

Despite these strong correlations, our ability to predict plant responses to temperature, alone and in conjunction with other environmental factors, remains imprecise. This may be because temperatures exceed some physiologically optimal temperatures (Asseng *et al*. 2013; Makowski *et al*. 2015; Wang *et al*. 2015). However, plants may also respond differently to temperature fluctuations than predicted from constant temperatures falling within the same temperature range (Yin and Kropff 1996; Karsai *et al*. 2008), as for instance, genotype-specific increases in leaf number for rice plants exposed to 30/22 °C day/night and night/day cycles compared to constant temperature regimes ranging from 22 °C to 32 °C (Yin and Kropff 1996). These observations underscore that predictions based both on mean and constant temperatures are limited. Further, the effect of non-stressing temperatures varies among cultivars (Karsai *et al*. 2013), and within a single cultivar, differing day-length or climate responses may also confound model prediction. For example, the same cultivar may show different thermal-time requirements, depending on planting date, location or growth conditions across years in the same location (Piper *et al*. 1996; Kumudini *et al*. 2014; Carter *et al*. 2017).

Thermal time accumulation describes an aggregate of underlying plant processes, and therefore may misrepresent some responses. Thermal units accumulate more slowly during cool growing seasons than warm ones, reaching a predetermined threshold later to predict flowering. This implies that all plant physiological rates decrease in tandem with temperature decreases. However, processes do not always slow under cool temperatures. The up-regulation of cryoprotective genes (Jaglo-Ottosen *et al*. 1998) and the circadian clock’s buffering to temperature changes (Rensing and Ruoff 2002) are just two examples. Therefore, models using thermal time could be improved or informed by incorporating or considering the molecular and genetic underpinnings driving plant response to temperature (Montesino-San Martin *et al*. 2014).

More mechanistic approaches decompose environmental influences into separate model processes (Welch *et al*. 2003; Kim *et al*. 2012; Brown *et al*. 2013; Zheng *et al*. 2013; Kiss *et al*. 2017). One such approach, in wheat, noted that the number of leaves produced before the reproductive transition decreased as the environmental signal’s strength increased (Jamieson *et al*. 1998a). Prolonged cold, vernalizing temperatures followed by longer days reduced the leaf number at which the transition occurred, while ambient temperature influenced the rate the leaves were produced (Brown *et al*. 2013). Modelling accumulation of *VRN3*, a key flowering gene, in response to vernalization and day-length cues, and as a function of thermal time, accurately predicted final leaf number and timing of flowering in the constant temperatures studied (Brown *et al*. 2013).

Now, temperature response mechanisms in the model plant *Arabidopsis thaliana* are becoming understood, providing opportunities to computationally test whether they could explain the thermal time response, as well as anomalous examples. Here, we used a modelling approach, building upon the approach (Brown *et al*. 2013) above, to assess the mechanistic basis of plant temperature response through accumulation of the key flowering regulator *FLOWERING LOCUS T* (*FT*). *Arabidopsis FT*, an orthologue of *VRN3* in wheat, is an integrator of environmental cues, its expression responding to photoperiod (day length), vernalization (prolonged periods of cold) and ambient temperature (Blázquez *et al*. 2003; Amasino 2010; Song *et al*. 2015). In most genotypes studied, high *FT* levels strongly correlate with flowering at an early developmental stage (i.e. low leaf number) (Krzymuski *et al*. 2015; Seaton *et al*. 2015; Kinmonth-Schultz *et al*. 2016). *FT* simulated as a function of day length and accumulated as a function of thermal time can accurately predict flowering in some conditions (Seaton *et al*. 2015). Under cool temperatures, *FT* is suppressed through the interaction of SHORT VEGETATIVE PHASE (SVP) and the FLOWERING LOCUS M (FLM)-β splice variant, and flowering is delayed, consistent with the concept of thermal time (Blázquez *et al*. 2003; Lee *et al*. 2013; Posé *et al*. 2013; Lutz *et al*. 2015; Kinmonth-Schultz *et al*. 2016; Sureshkumar *et al*. 2016). However, temperature fluctuations from warm to cool induce *FT* through induction of *CONSTANS* (*CO*), a chief transcriptional activator of *FT* (Schwartz *et al*. 2009; Kinmonth-Schultz *et al*. 2016). As there is no simple correlation between temperature decrease and *FT* level reduction, the linear accumulation of flowering gene products with thermal time may not adequately capture the influence of temperature on final leaf number, especially when temperature regimes shift over time.

In addition to direct temperature influence on *FT* gene expression, *FT* is expressed in the leaves, adding a second mechanistic layer through which temperature may impact whole-plant *FT* accumulation since the rate of leaf tissue production is strongly temperature-dependent (Parent *et al*. 2010). FT protein moves from the leaves to the shoot apex where it complexes with FD protein, a bZIP-type transcription factor, to induce the transition from leaf to floral production (Abe *et al*. 2005; Corbesier *et al*. 2007). The amount of FT protein perceived at the shoot apex likely depends on the amount of leaf tissue present. Since seasonal temperatures can be used to predict developmental rate and flowering, we aim to test the following hypotheses:

- The variability in observed thermal time accumulation at flowering could be explained by the accumulation of gene product (e.g. FT protein) and the capacity for its transcript production (e.g. leaf age and area) as a plant grows.
- Direct temperature influence on *FT* gene expression, like the direct influence of day length and vernalization, could improve model predictions in changing temperatures.

In this work, we used a mathematical modelling approach to assess the integrated influence of temperature acting at multiple scales of plant organization. To do so, we focused on *FT*, which correlates strongly to flowering time across a range of conditions (Corbesier *et al*. 2007; Salazar *et al*. 2009; Kinmonth-Schultz *et al*. 2016). We utilized earlier work in which we observed that *FT* could be both induced and suppressed by cool temperatures depending on whether constant or changing temperatures were applied (Kinmonth-Schultz *et al*. 2016). As *FT* is expressed in the leaves, and leaf growth is influenced by temperature, this provided us with an opportunity to determine the relative influences of *FT* transcriptional control versus whole-plant *FT* accumulation via leaf production.

**Figure 1.**
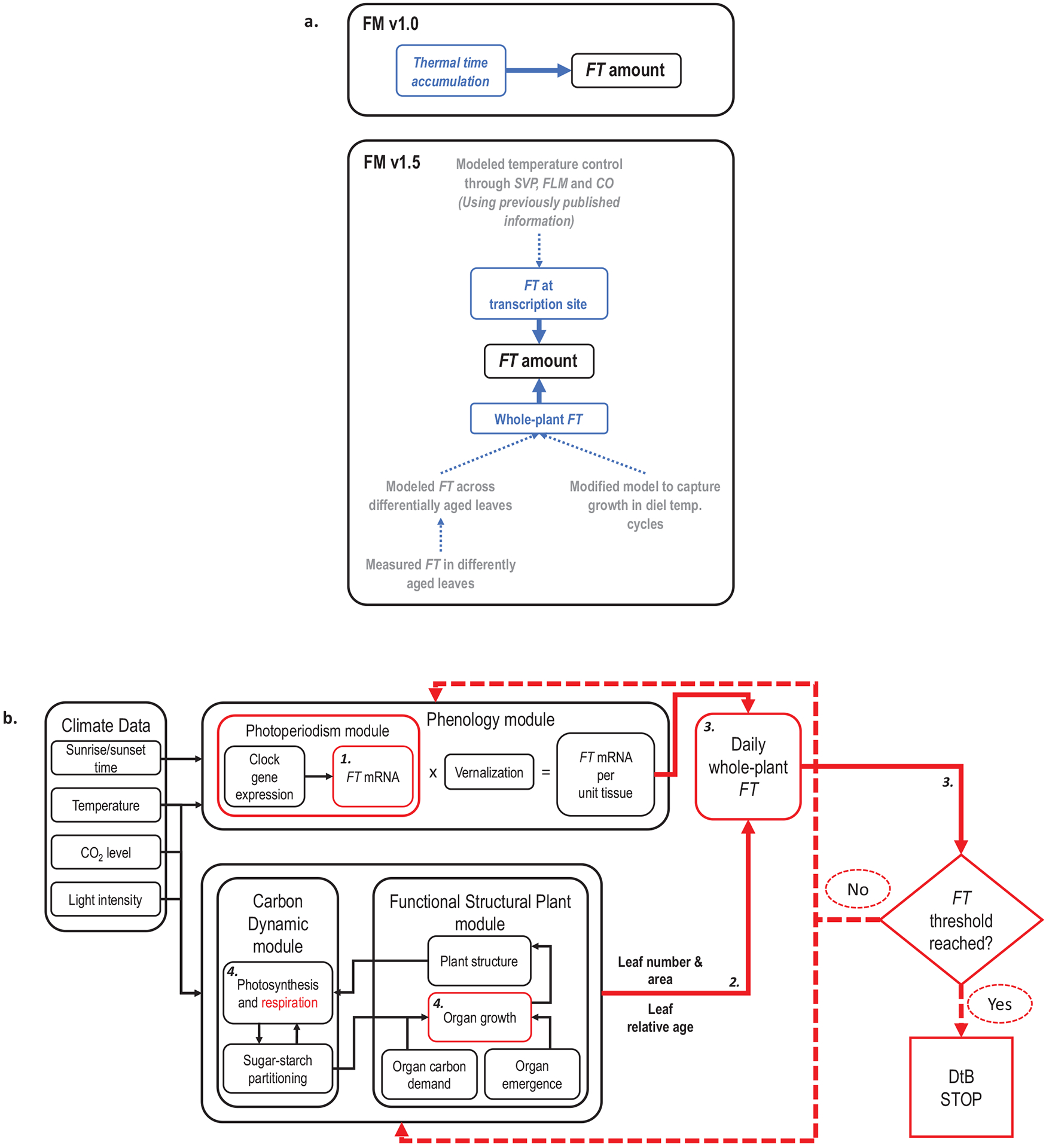
Overview of FM-v1.5. (A) Summary of how temperature influence on *FT* was modeled in FM-v1.0 and in FM-v1.5 (blue text) as well as the steps involved in modifying FM-v1.5 (gray text). (B) Schematic of Model FM-v1.5. Temperature (through SVP, FLM, and CO), day length, and the circadian clock regulate expression of *FT* in the Photoperiodism and Phenology modules per unit tissue. The leaf number and relative leaf age, outputs of the Functional Structural Plant module, are used to determine the capacity of each leaf to express *FT*, and leaf area is used to determine the amount of leaf tissue present. *FT* is summed across all leaves in a plant and added to the whole-plant *FT* from the previous time step. The model ceases leaf production and determines the days to bolt (DtB) when *FT* reaches a pre-set threshold set by using the leaf number for plants grown in long days (LD, 16-h light, 8-h dark) at 22°C, similarly to the way in which thermal units accumulate to a consistent value before a developmental transition occurs. Red illustrates where adjustments were made to the original model (FM-v1.0). The bold, italic numerals correspond to the numbers in the model description in text.

We first simulated *FT* transcriptional control by:

- Modelling *FT* suppression and induction by SVP, FLM and CO, based on review of previously published data, to impose dynamic control of *FT* over a range of temperature conditions (Fig. 1A).

Then, to simulate *FT* at the whole-plant level, we:

- Quantified the level of *FT* in differently aged leaves.
- Modelled the change in *FT* expression in leaves and plants over time.

*FT* transcript levels have neither been measured in leaves of different ages, nor been incorporated into flowering models, but doing so could improve our understanding of how day length and temperature impact *FT* to control flowering across developmental age. We incorporated these two models into the *Arabidopsis* Framework Model (FM-v1.0) and caused whole-plant *FT* to accumulate with the rate of leaf tissue production, which was modulated by temperature. Doing so required we modify FM-v1.0 to improve its capacity to simulate growth under dynamic temperature regimes (Fig. 1A).

Using this altered model, referred to as FM-v1.5, we:

- Explored the sensitivity of *FT* accumulation to both gene expression and leaf growth, as influenced by temperature.
- Asked whether *FT* could be used to predict flowering if accumulated to a threshold like thermal time accumulates.
- Explored the influence of both short- and long-term exposure to cool temperature on flowering; *FT* is induced and suppressed in these conditions, respectively.

Additionally, as FM-v1.0 had originally simulated *FT* accumulation as a function of thermal time, we compared our approach to the more traditional thermal-time approach used in FM-v1.0, which could accurately predict flowering in constant temperature conditions (Chew *et al*. 2014). Finally, we demonstrate situations in which our model fails to predict flowering and use those examples to discuss how our approach can serve as a framework for future work to quantitatively assess the role of *FT* compared with other molecular mechanisms.

## Model Description

### Overview

The *Arabidopsis* Framework Model (FM-v1.0; see Supporting Information—Fig. S1; Chew *et al*. 2014) combines plant growth and mechanistic flowering regulation for *Arabidopsis*. FM-v1.0 is run in two phases. In Phase 1, the timing of flowering is determined by thermal time accumulation (*T*(*t*) *− T*_*base*_, calculated hourly) in the Phenology module, with daytime temperature given more weight (Wilczek *et al*. 2009; Chew *et al*. 2012). Thermal time is modified by day length to produce Modified Photothermal Units (MPTUs) through mechanistic circadian- and day-length *FT* transcriptional regulation in the Photoperiodism module (Salazar *et al*. 2009). The number of days required to reach the MPTU threshold determines the stopping point of vegetative growth to mark the onset of flowering and is used as an input in Phase 2. In Phase 2, the climate parameters affect vegetative growth. Growth is determined by the rate of photosynthesis and carbon partitioning between roots and shoots (Carbon Dynamic module, Rasse and Tocquin 2006), and includes the rate of organ production as a function of thermal time, including production of individual leaves (Functional Structural Plant module, Christophe *et al*. 2008).

To modify FM-v1.0, we removed the thermal time accumulation used in Phase 1 of FM-v1.0 and instead incorporated mechanistic temperature influence on *FT* into the Photoperiodism module (Fig. 1). We maintained thermal time control over leaf tissue production in Phase 2 but modified the specific leaf area (SLA) and respiration components to improve the response of leaf growth to changing temperatures. Then, rather than running the model in two phases, we called the Phenology and Photoperiodism modules at each time step, considering their outputs *FT* gene expression per unit area (cm^−2^) of leaf tissue. We used the leaf number, age and area outputs at each time step to determine the relative *FT* produced by each leaf and summed the value of *FT* over the total leaf area of all leaves to get a whole-plant *FT* value. Below we detail our modifications (FM-v1.5, Fig. 1) along with a review of the data used to inform them. FM-v1.5 can be accessed at the following: https://fairdomhub.org/assays/1011.

### Data review and model modifications

#### 1. *FT* transcriptional regulation in changing temperatures regimes simulated through SVP, FLM and CO influence.

**Figure 2.**
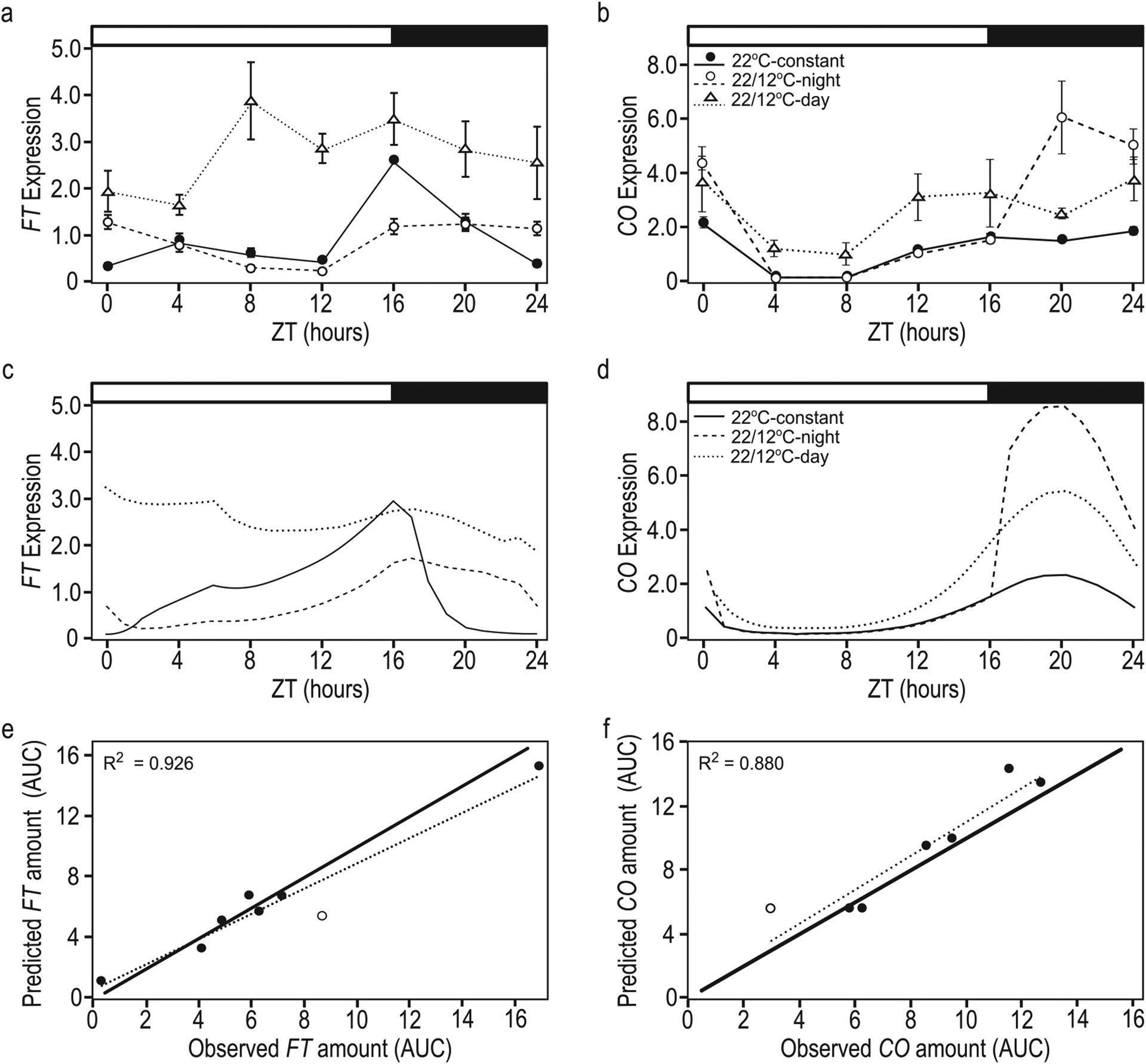
FM-v1.5 mimics general behaviours of *CO* and *FT* in response to temperature and can accommodate the overall change in amount across treatments. Observed (A, B) and predicted (C, D) diurnal patterns of *FT* (A, C) and *CO* (B, D) gene expression in warm-day, cool-night temperature-cycle (22/12 °C-night) treatments and in conditions in which the temperature dropped from 22 °C to 12 °C at dawn, then remained at the cooler temperature (22/12 °C-day) relative to the 22 °C-constant temperature control. The *y*-axis (A–D) is in nmol. The *x*-axis (A–D) is in zeitgeber time (ZT), and represents hours after dawn. The white and black bars represent light and dark periods, respectively. Error bars = 1 SE. If error bars are not visible, the SE is smaller than the height of the symbol. Correlation between predicted and observed results for *FT* (E) and *CO* (F), as calculated as the AUC 4 days after temperature treatments are imposed. Treatments include warm-day, cool-night cycles, drops to cooler temperatures at dawn, and growth from seed at constant temperatures. All treatment groups include 12, 17 and 22 °C. Dotted lines = correlation, solid lines = one-to-one line. Open circles are drop from 22 °C to 17 °C at dawn (C) growth from seed at 12 °C (F). Data from Kinmonth-Schultz *et al*. (2016) pooled and compiled in A and B (license to reproduce data obtained from John Wiley and Sons, #4601440806514).

To impose dynamic temperature regulation on *FT* transcription, we incorporated previous research on the control of *FT* via SVP, FLM and CO. These studies compared the amounts of transcript or protein relative to an endogenous control gene, the whole-plant concentrations of which are unknown. As we are concerned currently with the relative amounts across treatments, we, therefore, expressed their values in terms of leaf area (i.e. nmol cm^−2^). However, when multiplied by leaf area, the units become simply nmol, and we therefore express them as such throughout. Under LD, in 22 °C day, 12 °C night temperature-cycle conditions (22/12 °C-night), *FT* was suppressed at dusk compared to 22 °C constant temperatures (22 °C-constant) (Fig. 2A; Kinmonth-Schultz *et al*. 2016) likely through the action of SVP and the FLM-β splice variant, consistent with prior observations under constant temperatures (Blázquez *et al*. 2003; Lee *et al*. 2007, 2013; Posé *et al*. 2013). SVP protein levels increased shortly after exposure to cool temperatures (Kinmonth-Schultz *et al*. 2016), as did the amount of *FLM-β* compared to *FLM-δ* splice variants (Posé *et al*. 2013). FLM-β facilitates SVP binding, and SVP and FLM-β protein levels increased with decreasing temperatures (Lee *et al*. 2013). Both SVP and FLM-β were present at 23 °C; a transfer from 23 °C to 27 °C resulted in SVP decay that occurred within 12 h (Lee *et al*. 2013). Based on these observations, we used a single term: *SVP*_*p*_ to simulate the combined SVP and FLM-β behaviour representing ‘SVP/FLM activity’. Consistent with the observed behaviour of these proteins, we modelled the effect of SVP/FLM proteins (*SVP*_*p*_) to vary in response to temperature as shown below in equations (1.1–1.3).

(1.1) $$\text{SV}P_{p-\text{new}}(t)=\text{min}\{\text{SV}P_{\text{ceil}},{\text{b}}_{1}\cdot \text{exp}(-{\text{b}}_{2}\cdot \text{T}(\text{t}))\}$$

(1.2) $$\text{SV}P_{\text{ceil}}=\text{SV}P_{0}\cdot \text{exp}(-{\text{b}}_{3}\cdot {\text{d}}_{\text{FTL}})$$

*SVP_p-new_* is the rate of SVP protein (*SVP*_*p*_) production at time *t* (nmol h^−1^), *b*_1_ is an empirical parameter to set the *SVP* production rate that intercepts *T* = 0 °C, *T* is tissue temperature (°C) assumed to be equal to air temperature, *b*_2_ describes the degree SVP production decreases in response to a temperature increase and *t* is time of day. The influence of SVP may decline over time, as cool-temperature suppression of *FT* disappeared over a 2-week period [see Supporting Information—Fig. S2a]. To simulate this behaviour in *FT* [see Supporting Information—Fig. S2b and c], *SVP*_*ceil*_ is implemented as the ceiling of *SVP* production that declines relative to days post emergence of first true leaves (*d*_*FTL*_, equation (1.2)). *SVP*_0_ is the value of *SVP*_*ceil*_ prior to emergence of the first true leaf as determined at the time of sowing (nmol h^−1^). *b*_3_ determines the rate of decay over time (*d*_*FTL*_) and is unitless. *SVP_p-new_* is then input into a differential equation (1.3). Values and units of each coefficient are in Supporting Information—Table S1. To capture the suppression of *FT* at dusk, we set the SVP decay rate to be slightly lower than its production. This caused SVP to remain higher at 22 °C after a 12 °C night than in 22 °C-constant conditions, even after several hours at 22 °C [see Supporting Information—Fig. S2c]. The degradation rate is proportional to the relative amount of present SVP protein (*SVP*_*p*_) as represented by a rate constant (*v*_*SVP*_).

(1.3) $$\frac{\text{dSV}P_{p}}{dt}=\text{SV}P_{p-\text{new}}-(v_{\text{SVP}}\cdot \text{SV}P_{p})$$

In LD 22/12 °C-night, *FT* levels were higher at dawn co-inciding with higher *CO* mRNA and protein in cool nights (Fig. 2A and B) (Kinmonth-Schultz *et al*. 2016). While SVP/FLM activity may respond to absolute changes in temperature (Lee *et al*. 2007, 2013; Posé *et al*. 2013), *CO* accumulation was induced by rapid changes from warm to cool (Kinmonth-Schultz *et al*. 2016). The degree of temperature change is likely a factor, as a drop of 10 °C (22/12 °C-night) yielded more *CO* transcript accumulation than did a drop of 5 °C (22/17 °C-night) relative to 22 °C constant temperatures (Kinmonth-Schultz *et al*. 2016). This relationship was linear across the three treatments [see Supporting Information—Fig. S3a]. Based on these observations, we correlated *CO* mRNA induction (*K*_*T*_) linearly with the difference (*dT*) between the maximum and current temperatures (equation (1.4)). To determine *dT*, the model queried the temperature at each time step, and compared the current temperature against the previous maximum temperature. If higher, the current temperature was set as the new maximum temperature. *dT* could have been zero if there had been no decrease in temperature, so one was added to ensure *K*_*T*_ could not fall below one.

(1.4) $$K_{T}=1+(c_{1}\text{dT})\cdot \text{exp}(-c_{2}{\text{d}}_{\text{dT}})$$

Coefficient *c*_1_ is an empirical parameter that scales the degree at which *CO* induction changes with *dT*. The influence of a maximal temperature change faded over several days if the temperature remained cool over that time frame [see Supporting Information—Fig. S3b]. To account for this, *d*_*dT*_ is the time (days) since the maximal change in temperature occurred and *c*_2_ describes the decay in influence over time. *K*_*T*_ is used to modify the *CO* mRNA (*CO*_*m*_) amount to produce *CO*_*m-new*_ (equation (1.5)) to account for the temperature influence on *CO* through transcription (Kinmonth-Schultz *et al*. 2016).

With these modifications, simulated *CO* was induced (i.e. *CO*_*m-new*_) in response to a change to cool temperatures both during the day and at night like that observed (Fig. 2D). There was a strong relationship between the amount of simulated and observed *CO* transcript across treatments, calculated as the area under the curve (AUC, Fig. 2F); although, FM-v1.5 does not incorporate the *CO* suppression observed when plants are grown at constant 12 °C from seed (12 °C-constant) (Kinmonth-Schultz *et al*. 2016).

*CO*_*m-new*_ was used as an input to determine CO protein (*CO*_*p*_) as in Chew *et al*. (2014), as shown below (equation (1.6)). CO protein degradation occurred only in the dark period (*L*_1_ = Light period).

(1.5) $$CO_{m-\text{new}}=CO_{m}\cdot K_{T}$$

(1.6) $$\frac{\text{dC}O_{p}}{\text{dt}}=v_{CO_{m}}\left(CO_{m-\text{new}}\right)-v_{CO_{p}}\frac{CO_{p}}{k_{CO_{p}}+CO_{p}}\left(1-L_{1}\right)$$

As several factors converge to regulate *FT* at its promoter (Bratzel and Turck 2015), we hypothesized that the SVP/FLM complex and CO acts competitively at the *FT* promoter, with CO overcoming suppression by SVP/FLM at night when its levels are high. The Photoperiod module in FM-v1.0 (Chew *et al*. 2014) describes the direct relationship between transcription of *FT* mRNA (*FT m*) and CO protein (equation (S1.1)). We modified this equation to incorporate the interaction between CO and SVP/FLM using a modified Michaelis–Menten function for competitive inhibition (Segal 1976), such that the *K* of *FT* induction by CO was influenced competitively by SVP/FLM activity as below.

(1.7) $$\frac{\text{dF}T_{m}}{\text{dt}}=L_{2}\cdot \left(V_{\text{CO}}\frac{CO_{p}}{K_{\text{CO}}\left(1+\frac{\text{SV}P_{p}}{K_{\text{SVP}}}\right)+CO_{p}}-V_{\text{FT}}\frac{FT_{m}}{K_{\text{FT}}+FT_{m}}\right)$$

The uppercase *V* and *K*, and lowercase *v* and *k* are Michaelis–Menten constants either describing the *FT* synthesis rate as influenced by CO protein (*CO*_*p*_), SVP/FLM activity (*SVP*_*p*_) or *FT* degradation. We observed *CO* and *FT* induction when the temperature dropped both at dawn and dusk (Kinmonth-Schultz *et al*. 2016), like previous observations (Thines *et al*. 2014). However, observed daytime *CO* induction was lower than nighttime induction while daytime *FT* induction was higher (Fig. 2A and B). The daytime CO protein production captured in equation (1.6) was not enough to capture the daytime behaviour of *FT* in FM-v1.5 in response to daytime drops in temperature. While dusk regulation of *FT* is well understood (Song *et al*. 2015), the factors influencing morning *FT* induction are only beginning to be elucidated (Song *et al*. 2018). To capture the observed behaviour, we increased *FT* transcriptional sensitivity in the morning (*L*_2_) using a switch function that relied on a model component that peaks around dawn, specifically the circadian clock component, *LHY*, from the Photoperiodism module [see Supporting Information—Fig. S4].

With these modifications incorporated into the model, CO induced higher *FT* at dawn when temperatures dropped at night (22/12 °C-night), and higher *FT* throughout the day when temperatures dropped just after dawn (22/12 °C-day) as previously observed (Fig. 2C). Our simulation in which SVP/FLM activity decay was slower than production caused residual SVP/FLM activity at dusk [see Supporting Information—Fig. S2c], suppressing *FT* at dusk as observed (Fig. 2C, 22/12 °C-night). When we removed SVP influence to mimic a *svp* mutant, dusk *FT* suppression disappeared as observed [see Supporting Information—Fig. S5]. However, morning levels of *FT* in a simulated *svp* mutant were higher than observed, perhaps because we increased morning *FT* transcriptional sensitivity to CO. This high induction was necessary to approximate the observed behaviour of *FT* in wild-type, especially the very high daytime induction observed when the temperature drop occurred at dawn (22/12 °C-day). Therefore, while SVP, FLM and CO may act competitively to regulate *FT*, additional mechanisms likely increase *FT* transcription, perhaps in a time-dependent manner, despite the presence of SVP and FLM repression in cool temperatures.

For flowering to occur, favourable conditions must occur over several days (Corbesier *et al*. 2007; Krzymuski *et al*. 2015; Kinmonth-Schultz *et al*. 2016). Our aim was to approximate *FT* behaviour within a day and through time. Observed *FT* suppression at dusk in 22/12 °C-night conditions occurs by Day 2 of the temperature-cycle treatment relative to the 22 °C-constant control [see Supporting Information—Fig. S2a]. This was true with FM-v1.5 as well, although *FT* levels continue to decline until Day 4 [see Supporting Information—Fig. S2b]. Over 2 weeks, the increase in dusk *FT* levels in 22/12 °C-night conditions relative to the 22 °C-constant control was similar between observed and simulated data [see Supporting Information—Fig. S2b]. In sum, FM-v1.5 can accommodate the wide range in *FT* transcribed across temperature treatments (Fig. 2E), and *FT* behaves similarly over time to that observed, allowing us to explore the temperature influence on *FT* expression and flowering in LDs.

#### 2. Incorporating *FT* as a function of leaf and plant age.

**Figure 3.**
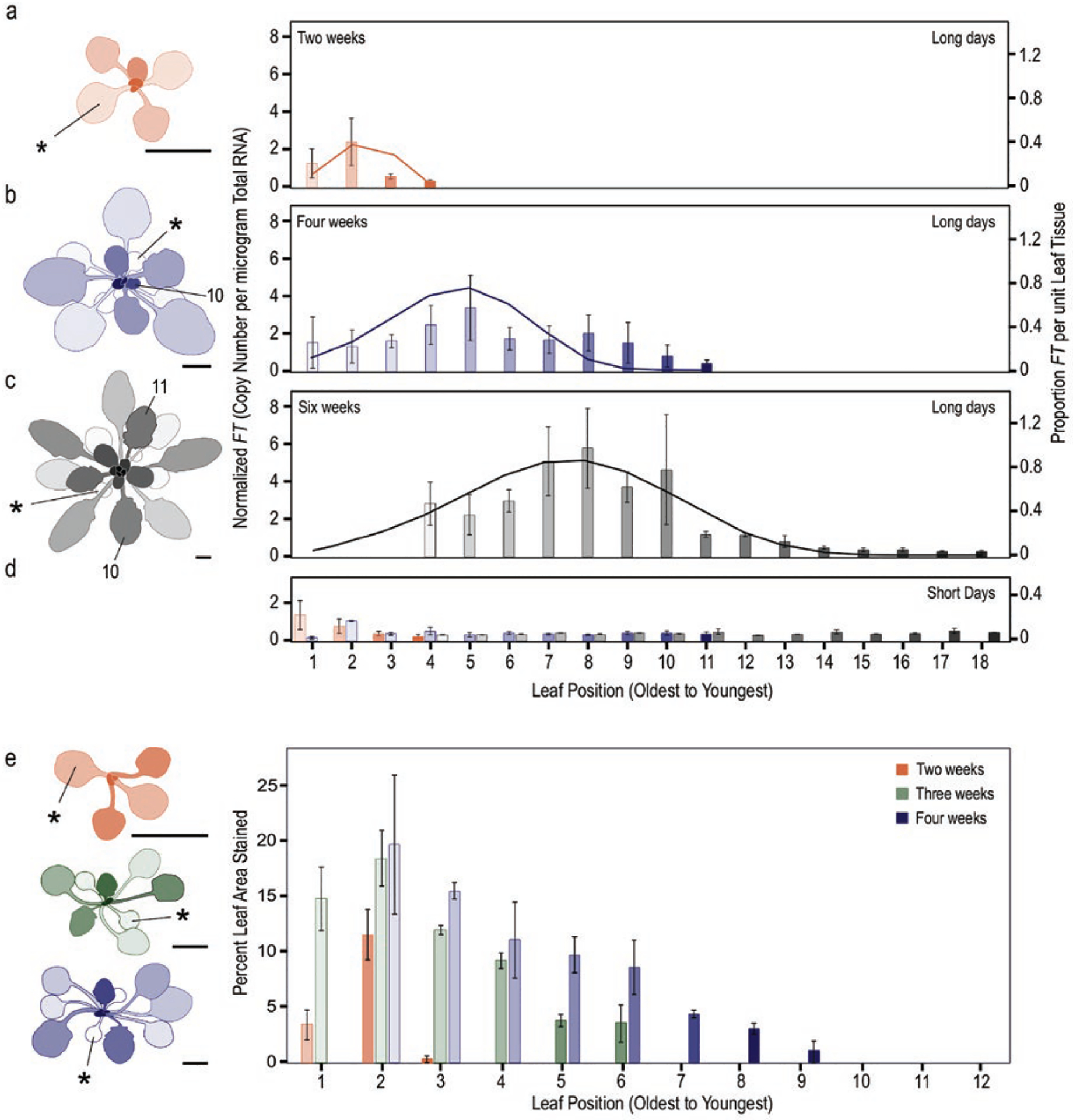
*FT* expression declines in later produced leaves. Leaves of short-day-grown plants, that had not yet transitioned to flowering, aged 2 (A), 4 (B) and 6 (C) weeks old were exposed to long days or short days (D) for 3 days, then harvested at 16 h after dawn on the third day to determine *FT* amount per leaf. The colours in (D) correspond to the colours and ages in panels (A–C). *FT* levels were determined by absolute copy number and normalized within a replicate. The simulated proportion of *FT* per unit leaf tissue (cm^−2^, solid lines) for each plant age is shown. This value was used in FM-v1.5 as a modifier to adjust the amount of *FT* produced by each leaf. Percent of the leaf area showing staining in *pFT:GUS* plants (E). For all, the two cotyledons and first two true leaves were pooled for each sample as they emerge in pairs. The youngest leaves, just emerging at the apex (1–2 mm in length) were also pooled. Older leaves in the 6-week-old plants failed to yield 2 μg total RNA and were excluded. For each plant inset, asterisk indicates one of each cotyledon pair (*). The 10th and 11th leaves to emerge are labelled. The shading of the bar graphs (light to dark) indicates leaf age (oldest, first to emerge, to youngest) and corresponds to the shading in the plant insets. Scale bars = 0.5 cm.

As our aim was to simulate whole-plant *FT* accumulation with leaf growth, we needed to ascertain whether *FT* expression was consistent across all leaves produced and across all ages. SD-grown (8 h light, 16 h dark) plants aged 2, 3, 4 and 6 weeks exposed to LDs for 3 days to temporarily induce *FT* as in Corbesier *et al*. (2007) showed differing capacities to express *FT*. (SDs cause a delay in the transition to reproduction in *Arabidopsis*, and the lines used do not transition to reproduction in these time frames, as in 8-week-old plants in Corbesier *et al*. (1998)). In young plants, even very young leaves expressed *FT*. This capacity changed as plants age (Fig. 3; see Supporting Information—Fig. S6). Newer leaves in older plants seemed to lose capacity to express *FT* (Fig. 3; see Supporting Information—Fig. S6), even those that had expanded. Considering *FT* copy number per unit leaf tissue, the amount of *FT* increased from the cotyledons to the older true leaves in plants aged 2, 4 or 6 weeks in LD, declining again in more newly produced leaves (Fig. 3A–D; see Supporting Information—Fig. S6). In 6-week-old plants, several of the newest leaves showed *FT* expression levels comparable to those in SD conditions (Fig. 3C and D), even in leaves that had expanded to sizes that were comparable or larger than leaves of plants expressing *FT* at a younger age. Using *pFT:GUS* (Takada and Goto 2003) plants to assess the location of *FT* on a per area basis, *FT* increased from cotyledons to the true leaves (number 2, Fig. 3E) then declined again in more newly produced leaves. For several of the true leaves on the younger plants, *pFT:GUS* staining spanned the tip to the base of the leaves [see Supporting Information—Fig. S6a and b]. In 6-week-old plants most leaves sampled showed staining only at their edges regardless of leaf size [see Supporting Information—Fig. S6d]; remnant staining appeared to occur throughout the blade of older leaves. Both analyses suggest that in addition to leaves losing the capacity to express *FT* as they age, newer leaves in older plants also lose the capacity to express *FT*. We incorporated the relationship describing *FT* expressed per unit leaf tissue into FM-v1.5. Future work to address the mechanisms for this change with developmental age is needed.

To simulate the proportion of *FT* per unit tissue (*FT*, nmol cm^−2^) of each leaf, we used a beta function (Yin *et al*. 1995) based on relative leaf age (*r*), beginning with the youngest emerged leaf as leaf one.

(2.1) $$\beta_{\text{FT}}=\text{max}\left(0,\beta_{\text{FTmx}}{\left[\left(\frac{\text{r}}{{\text{R}}_{\text{opt}}}\right){\left(\frac{{\text{R}}_{\text{crit}}-\text{r}}{{\text{R}}_{\text{crit}}-{\text{R}}_{\text{opt}}}\right)}^{\left(\frac{{\text{R}}_{\text{crit}}-{\text{R}}_{\text{opt}}}{{\text{R}}_{\text{opt}}}\right)}\right]}^{\text{e}}\right)$$

*β*_*FT*_ yields a value between zero and no more than one but can be lower depending on plant age as measured by leaf number. *β*_*FTmx*_ scales, based on total leaf number, the maximum value that can be attained by a leaf of a single plant, *R*_*opt*_ is the relative age at which that maximum value is attained, *R*_*crit*_ is the oldest leaf that can express *FT* and *e* described the steepness of the curvature. This function caused the dependent variable to oscillate if the independent variable spans a broad range. To avoid this behaviour, we set *β*_*FT*_ to be zero below and above the relative ages where *β*_*FT*_ first attains a minimum. *β*_*FTmx*_ and *R*_*opt*_ are dependent on the total number of leaves on a plant (*l*), as described below, avoiding the need to reparameterize for plants of different ages. *g*_1_ and *g*_2_ are empirical coefficients.

(2.2) $$\beta_{\text{FTmx}}=1-\left(\frac{g_{1}}{l}\right)$$

(2.3) $$R_{opt}=g_{2}\cdot l$$

#### 3. Determining whole-plant *FT* levels and accumulating *FT* to a threshold.

To link *FT* transcript accumulation to leaf tissue production, the Phenology module was called at each time step. We considered the output of the Phenology module to be the amount of *FT* produced per unit leaf area (*FT*_*leaf*_, nmol cm^−2^) in order to scale for total *FT* at the whole-plant level. The whole-plant *FT* level (*FT*_*plant*_) was determined by a summation of all *FT*_*leaf*_ adjusted by individual leaf area (*LA*, cm^2^) and the capacity of each leaf to express *FT* (*β*_*FT*_, unitless modifier) as *FT* induction is dependent on light intercepted by the leaf.

(3.1) $$FT_{\text{plant}}=\overset{l}{\underset{n=1}{\sum }}\left(FT_{{\text{leaf}}_{n}}\cdot LA_{n}\cdot \beta_{FT_{n}}\right)$$

At each time step, *FT*_*leaf*_ (nmol cm^−2^) was determined for each leaf, summed across all leaves and added to the value from the previous time step to determine whole-plant *FT* levels on a per area basis, which can be converted to *FT* per unit mass using the leaf mass ratio as needed. Such *FT* accumulation was consistent with the observation that several days of *FT* induction are needed to induce flowering (Corbesier *et al*. 2007; Krzymuski *et al*. 2015; Kinmonth-Schultz *et al*. 2016). To predict flowering, the model ran until a threshold level of *FT* was reached at the whole-plant level (*FT*_*thrsh*_). We set the threshold as the value of *FT* accumulated when plants reached 15 and 8 leaves [see Supporting Information—Table S1], which was the nearest whole number to the average leaf number at bolt for Columbia-0 (Col-0) and Landsburg *erecta* (L*er*), respectively, grown in LD 22 °C-constant conditions (Kinmonth-Schultz *et al*. 2016). We maintained the strain-specific parameters for rate of emergence and leaf initiation from FM-v1.0, as they were comparable to our results (Fig. 5A), but added a 7-day delay after initiation of the final leaf to improve the fit across strains at 22 °C. This was to account for the time between initiation of the leaf primordia as modelled (Christophe *et al*. 2008) and growth of a visible bolt, counted when the stem below the bolt head was 2 mm in length (Kinmonth-Schultz *et al*. 2016).

Here, we considered whole-plant levels of *FT*, rather than the amount reaching the shoot apex, consistent with typical molecular studies that consider gene expression at the level of the whole plant (Imaizumi *et al*. 2003; Valverde *et al*. 2004). Through this approach, we inherently assumed that the rates of translocation of *FT* to the shoot apical meristem and the proportion of whole-plant *FT* perceived at the shoot apex are similar across treatments. All other treatments were run to this threshold under the assumption that it remains conserved under different growing temperatures.

In FM-v1.0, the development rate towards flowering, as influenced by *FT* amount and photoperiod, was limited below and above two critical day lengths (10 and 14 h) using a different parameter set for each photoperiod (Chew *et al*. 2014).

(3.2) $$\text{Photoperiod}=A+B\left[\frac{C^{n}}{C^{n}+{\left(\text{FTarea}\right)}^{n}}\right]$$

We removed this function and considered direct *FT* accumulation. Determining the absolute amount of *FT* required to induce flowering and whether there are threshold levels of transcription, below and above which flowering time is unaffected, will be a useful future study. We maintained the vernalization component from FM-v1.0 to maintain model flexibility, as vernalization should modify overall levels of *FT* (Helliwell *et al*. 2006; Searle *et al*. 2006). This value fell between zero and one and now modified the levels of *FT* produced within the Phenology model rather than modifying the thermal unit accumulation rate.

#### 4. Adjusting FM-v1.0 leaf area response to changing temperature regimes.

FM-v1.0 was parameterized for constant temperatures. It captured the leaf areas of plants exposed to different constant temperatures but simulated larger areas for plants grown in temperature regimes that change over time than the constant-temperature control [see Supporting Information—Fig. S7a]. Conversely, observed plants accumulated similar biomass, but a lower SLA (m^2^ g^−1^) under changing temperatures relative to a constant-temperature control (Pyl *et al*. 2012). Therefore, in FM-v1.5, we adjusted the SLA and respiration components to improve the relationship among leaf areas across changing temperature conditions (described below).

The larger leaf area in FM-v1.0 under a temporal shift in temperature regime, specifically a drop in temperature, occurred for two reasons. First, SLA was observed to decrease with increasing thermal time (i.e. developmental time, Christophe *et al*. 2008). When incorporated into FM-v1.0, this relationship caused simulated SLA to be lower in warmer conditions because development was faster [see Supporting Information—Fig. S7b and c] although all treatments began at a similar biomass. Second, FM-v1.0 related maintenance respiration to temperature using the Arrhenius function, causing respiration to be lower under cooler temperatures. Under warm daytime temperatures, plants simulated in changing temperatures accumulated the same amount of stored carbon as the control [see Supporting Information—Fig. S7d]. However, once shifted to cooler temperatures, the lower simulated maintenance respiration rate [see Supporting Information—Fig. S7e] left a larger stored carbon pool that could be used for growth, causing larger leaves in FM-v1.0.

Respiration, carbon storage or growth may be altered by temperature in ways not captured in the model. In cold-tolerant woody species, respiration of stem cuttings increased near freezing, rather than following the trend predicted by the Arrhenius function, as did the pool of non-structural carbohydrates (NSCs) (Sperling *et al*. 2015). Respiration may also increase at more moderate temperatures in cases where freezing tolerance is induced, as in *Arabidopsis* at 16 °C in light with a low red/far-red ratio (Franklin and Whitelam 2007). In chrysanthemum, cool nighttime temperatures decreased leaf area while increasing dry weight, by increasing stored starch (Heinsvig Kjær *et al*. 2007). FM-v1.0 did not incorporate these complexities nor consider sinks for carbon other than growth, such as NSCs. Therefore, to simulate the relative relationships in leaf area across the temperature conditions needed for our study, we removed the temperature sensitivity of maintenance respiration and adjusted the SLA (m^2^ g^−1^) to decline with decreasing temperature using observations from Pyl *et al*. (2012) [see Supporting Information—Fig. S8]. This resulted in lower SLA across treatments than the control when there was a temporal shift to cooler temperatures [see Supporting Information—Fig. S7f]. A more accurate representation of respiration and carbon pools should be incorporated into future models to improve plant growth predictions in a range of temperature conditions.

### Model parameterization

To entrain the diurnal *FT* and *CO* patterns, we incorporated previously published data from three different treatment types all in 16-h photoperiods grown at ~60 μmol m^2^ s^−1^ photon flux density: warm-day (22 °C), cool-night (12 or 17 °C) temperature cycles, in which the temperature change occurred at dusk (24 wild-type replicates, six including 17 °C, and five including the *svp* mutant line); constant warm (22 °C) temperatures shifting to constant cool (12 or 17 °C) temperatures at dawn (eight and three replicates, respectively); and growth at 12 and 17 °C from seed (three replicates each) (Kinmonth-Schultz *et al*. 2016) (pooled data shown in Fig. 2A and B, see Supporting Information—Figs S3a and S9a). Except for where ‘growth from seed’ is specified, plants were moved to temperature treatments after 1 week in 22 °C-constant temperature conditions. In all instances, growth from seed at 22 °C was used as the control. The temperature-cycle harvests including 17 °C spanned 2 days. An ANOVA comparison of models including and excluding *day* as a factor, showed no difference. The days were counted as separate replicates for model training. *FT* and *CO* gene expression were pooled across all replicates within a treatment and normalized across treatments to the mean peak *FT* expression (ZT 16) and mean peak *CO* expression (ZT 16 and 20 mean) in the 22 °C control. Parameter values for change in *CO* induction and *SVP/FLM* activity over a period of days were determined using experiments with four replicates each (Kinmonth-Schultz *et al*. 2016) (data shown in Supporting Information—Figs S2a and S3b). As we were interested in the cumulative influence of *FT*, we assessed model fit and performance in three ways: (i) minimizing RMSE between observed and predicted gene expression profiles over the 24-h harvest period (14 days after sowing), (ii) comparing observed and predicted amounts of *CO* and *FT* as calculated as the AUC 14 days after sowing and (iii) maintenance of gene expression patterns through time.

## Experimental Methods

### Plant growth conditions

For all previously published and new plant growth experiments used in this work, seeds were sewn onto soil (Sunshine #3 Mix; Sun Gro Horticulture) containing Osmocote Classic time-release fertilizer (Scotts) and Systemic Granules: Insect Control (Bionide), cold and dark stratified at 4 °C for 4 days, then moved to temperature treatments in either long-day (LD, 16-h light, 8-h dark; light intensity = ~60 μmol m^2^ s^−1^) or short-day (SD, 8-h light, 16-h dark; light intensity = ~140 μmol m^2^ s^−1^) conditions as previously described (Kinmonth-Schultz *et al*. 2016).

To explore the effect of conditions in which *FT* is suppressed (Fig. 6), we grew Col-0 at 12 °C from seed (12 °C-constant) or in 22 °C for 1 week then moved plants to 12 °C within 2 hours after dawn (22/12 °C-day). To explore the influence of short-term temperature fluctuations (Fig. 7), we grew Col-0 plants in SDs for 2 weeks, opposed to the 1 week in experiments used to parameterize mechanistic temperature control of *FT*, to ensure they had two to three true leaves and were competent to flower. We then exposed them to 12 °C in LDs for 2, 4 or 6 days (12 °C-2d, -4d or -6d). After treatment, plants were moved to warm, LD conditions. Control plants were moved directly to warm, LD conditions at 2 weeks. The chambers used for the warm conditions averaged 24 °C. This was incorporated into simulations.

To determine the capacity of leaves of different ages to express *FT*, we grew Col-0 (for RNA isolation) and *pFT:GUS* (Takada and Goto 2003) plants for 2, 3 (*pFT:GUS* only), 4 or 6 weeks in SDs—conditions in which flowering is delayed and rosette leaf production is prolonged. They were moved to LD conditions 3 days prior to harvest, which is adequate time to see day-length-specific induction of *FT*, but before the transition to reproduction is well underway (Corbesier *et al*. 2007; Krzymuski *et al*. 2015; Kinmonth-Schultz *et al*. 2016), or retained in SD as a control.

### Determining *FT* expression levels in leaves of different ages (RNA and GUS analysis)

We used analysis of *FT* RNA levels and *pFT:GUS* signal to determine the capacity of differently aged leaves to express *FT*. For RNA analysis: leaves of the same relative age were pooled from at least six plants. The cotyledons and first true leaves emerge in pairs, and the leaves of a pair were not separated for this analysis. Small, newly emerged leaves (1–2 mm) were also not distinguished separately, but rather harvested as a group. We isolated RNA (illustra RNAspin, GE Healthcare, Chicago, IL, USA), equalizing the isolate to ensure similar starting material across samples for RT-qPCR. We excluded any samples under 2 μg total RNA, which were primarily those of older leaves. The transcript copy number in each sample was determined using a serial dilution generated from an *FT* fragment spanning base pairs 1900 to 2135 from the transcriptional start site in exons 3 and 4. The fragment was amplified by RT-PCR and concentrated using ethanol purification. Fragment copy number was determined using the following equation: $\text{Copy}\;\text{number}=C\left(\frac{NA}{M}\right)$ where Copy number = number of molecules per μL, *C* = concentration of the purified PCR product (g μL^−1^), *M* = molecular weight of the purified region, *NA* = Avogadro’s number = 6.023 × 10^23 molecules per mole (Fernández-Aparicio *et al*. 2013). The concentration was determined using a nanodrop (NanoDrop Lite, Thermo Scientific, USA), and then diluted using an eight-step, 1:10 serial dilution. The serial dilution was run along with the plant sample during qPCR and used to develop a linear relationship between copy and cycle number. Replicates were normalized by the replicate average.

For GUS stain image analysis: at least three whole plants of each age (2, 3 or 4 weeks old) were harvested concurrently at dusk. The plants were incubated in chilled 90 % acetone for 45 min. Prior to GUS staining, we rinsed the plants in water. Next, we immersed the plants in a GUS staining solution composed of 50 mM NaPO_4_, pH 7.4, 2 mM X-gluc (Gold Biotechnology, St. Louis, MO, USA), 0.5 mM K_3_Fe[CN]_6_ and 0.5 mM K_4_Fe[CN]_6_. We vacuum infiltrated the plants three times for 10 min, as previously described (Sieburth and Meyerowitz 1997). We incubated the plants at 37 °C in the staining solution for 48 h. Next, to clear the tissue, we incubated the plants at 4 °C for 30 min at each step of the following series: 30 % ethanol, fixing solution (50 % ethanol, 5 % acetic acid and 3.7 % formalde-hyde in water), 80 % ethanol and 95 % ethanol. Plants remained in 95 % ethanol overnight. We then photographed the plants, in 95 % ethanol, using a dissecting scope (Leica S8APO). Plants that were too large to be captured in one image were photographed piecewise, and the images were integrated using the GNU Image Manipulation Program (GIMP) (Kimball *et al*. 1995). During *FT* expression quantification, ImageJ struggled to distinguish between the blue of the GUS staining and the unstained leaf tissue; to more precisely quantify *FT* expression, we used GIMP to manipulate photos by tracing any locations stained blue, so they could be detected and analysed using ImageJ software (Rasband 1997). We then used ImageJ to quantify both the total area of *FT* expression and the total area of each leaf. Finally, we calculated the percentage of leaf area for each leaf that displayed *FT* expression. We have included both the traced and untraced versions here [see Supporting Information—Fig. S6]. The results for leaves on a single plant that were the same age (i.e. cotyledons and first true leaves) were averaged to obtain a single value for the pair. Then, values for leaves of the same age were averaged across all plants within each age group (i.e. 2, 3 or 4 weeks).

### Statistical analysis and experimental controls

Statistical analysis on final leaf number and flowering time for the short-term temperature experiment was done using Generalized Estimating Equation (Carey 2015) in R Statistical Computing Software (v3.1.1; R Core Team 2015), which accounts for missing values and heteroscedasticity of residuals. The Robust *Z* and standard error were used to calculate *P*-values and 95 % confidence intervals (CIs) of the difference between the means. Plants were organized into groups of four by similarity of size and leaf number then split into the four temperature treatments to avoid the confounding effects of size. These groups were accounted for in the statistical model. The date of visible bolt was recorded when the stem below the bolt head was 2 mm in length. Floral stems were removed before rosette leaves were counted as stem height differed across temperature treatments and rosette leaves were counted blindly (without knowledge of the temperature treatment) to avoid bias. The experiment was replicated twice, with ‘replicate’ also being included as an effect in the model. Normality of residuals was determined through observation of normal Quantile-Quantile plots and the Generalized ESD Many-Outlier Procedure (Rosner 1983), resulting in detection of three outliers: one in days to bolt (2d in 12 °C treatment), and two in rosette leaf number (4d in 12 °C treatment). Justification was found to remove the latter, as they were counted by a different researcher and may have included leaves produced by auxiliary meristems, no justification was found to remove the former and thus it was not removed. Student’s *t*-tests (*stats* package, R Core Team 2015) were used to compare the leaf number of plants grown in 24 °C (control) or 12 °C temperature conditions after 2, 4 and 6 days in temperature treatments. Treatment effects were considered significant when the *P*-values fell below 0.05 and the CI did not contain zero. For the *t*-tests, *P*-values and CI were lowered from these values for multiple comparisons using the Bonferonni correction.

## Results

### Assessment of *FT* accumulation in FM-v1.5 across temperatures in response to gene expression or leaf growth

We began by assessing the rates of *FT* accumulation and the relative temperature influences of both gene expression and leaf development. We compared the total *FT* accumulated 9 days post emergence, equivalent to 1 week in temperature treatments, considering (i) influence of temperature on gene expression only (GE), (ii) *FT* accumulated with leaf tissue production as influenced by thermal time, temperature influence on gene expression excluded (LTP) and (iii) gene expression changes incorporated with leaf tissue production (LTP+GE, full FM-v1.5 model). The influence of age on a leaf’s capacity to express *FT* is incorporated into both the LTP and LTP+GE model variants.

**Figure 4.**
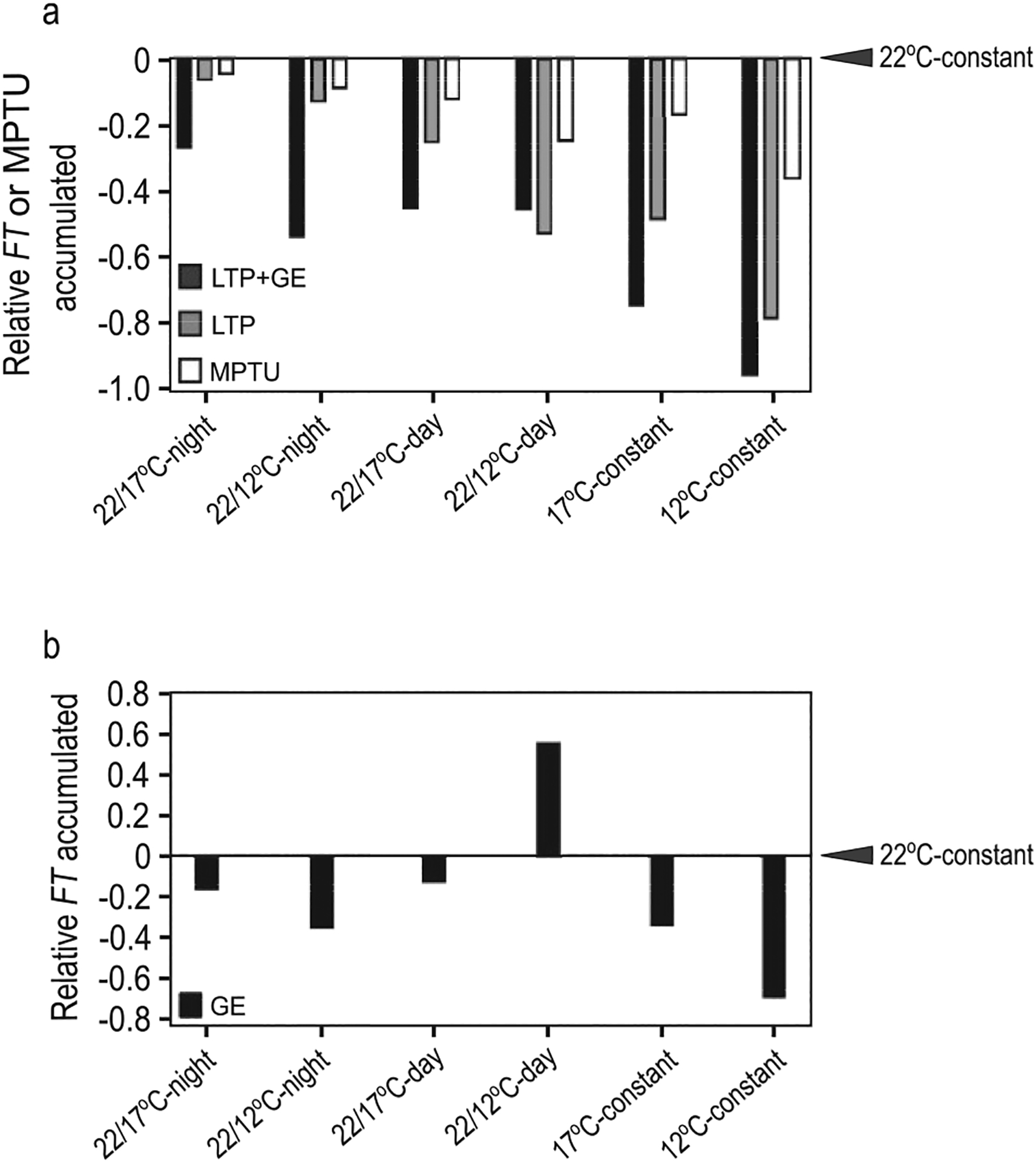
(A, B) Whole-plant *FT* accumulation influenced by temperature in changing and constant cool-temperature conditions, differs more strongly from the 22 °C control than does accumulated MPTUs. Total *FT* accumulated in constant and changing temperature conditions relative to 22 °C constant temperatures (indicated by arrowheads) 9 days post emergence, equivalent to 1 week in changing temperature treatments. (A) LTP+GE: *FT* accumulation in full FM-v1.5 model, i.e. temperature affects *FT* gene expression though CO and SVP/FLM as well as through leaf tissue production; LTP: *FT* accumulation only with leaf tissue production as influenced by temperature, temperature influence on *FT* gene expression excluded; MPTU: accumulated Modified Photothermal Units from FM-v1.0. Here, daytime and nighttime temperatures are given equal weight. (B) GE: *FT* accumulation considering only influence of temperature on *FT* gene expression, decoupled from leaf production. *22/12* or *17 °C-night* indicates warm-day, cool, night cycles, *22/12* or *17 °C-day* indicates treatments in which the temperature drop occurred at dawn, then remained cool for the duration of the experiment, constant indicates temperatures remained constant from seed.

When considering LTP+GE, total *FT* declined relative to the 22 °C-constant control, with increasing exposure times to cool temperature as would be expected from leaf area changes (Fig. 4A). When only transcriptional changes were considered (GE), *FT* accumulated at a faster rate than the control for some treatments (i.e. a drop in daytime temperature, Fig. 4B). For treatments in which *FT* accumulated more slowly than the control, as in 12 °C-constant, the relative difference from the control was less extreme than in LTP+GE. For comparison, we explored the relative difference in accumulated MPTUs, which control flowering time in FM-v1.0, over this time frame. MPTUs across treatments differed to a lesser degree than accumulated *FT* transcript in LTP+GE, even when nighttime temperatures carried the same weight as daytime temperatures (Fig. 4A).

To assess the influence *FT* transcriptional changes due to temperature have on whole-plant *FT* levels, we used the LTP model variant, meaning that temperature influenced *FT* only through leaf production modulated by thermal time and did not affect *FT* gene expression levels. Total *FT* accumulation in the warm-day, cool-night temperature-cycle treatments was higher in the LTP variant than the LTP+GE variant although it was still lower than the *FT* accumulated in the constant-temperature control as leaf production and expansion was slower (Fig. 4A). When the daytime temperature dropped from 22 °C to 12 °C (22/12 °C-day) *FT* accumulated more quickly in LTP+GE than in LTP. Taken together, we find that whole-plant *FT* (LTP variant) accumulates in a manner like that of thermal time, in that its rate of accumulation is altered in response to temperature. Incorporating temperature influence on *FT* gene expression (LTP+GE) can accelerate or decelerate whole-plant *FT* accumulation depending on the type of temperature fluctuations experienced.

### Assessing capacity of *FT* accumulated to a threshold in FM-v1.5 to predict flowering

We next asked how well *FT* accumulation could predict flowering, and whether incorporating temperature influence through transcriptional control of *FT* improved model predictions when there is a temporal shift in temperature regime. To assess this, we simulated experiments for plants grown in warm-day, cool-night temperature cycles (Kinmonth-Schultz *et al*. 2016) as plants often experience such temperature fluctuations in nature.

**Figure 5.**
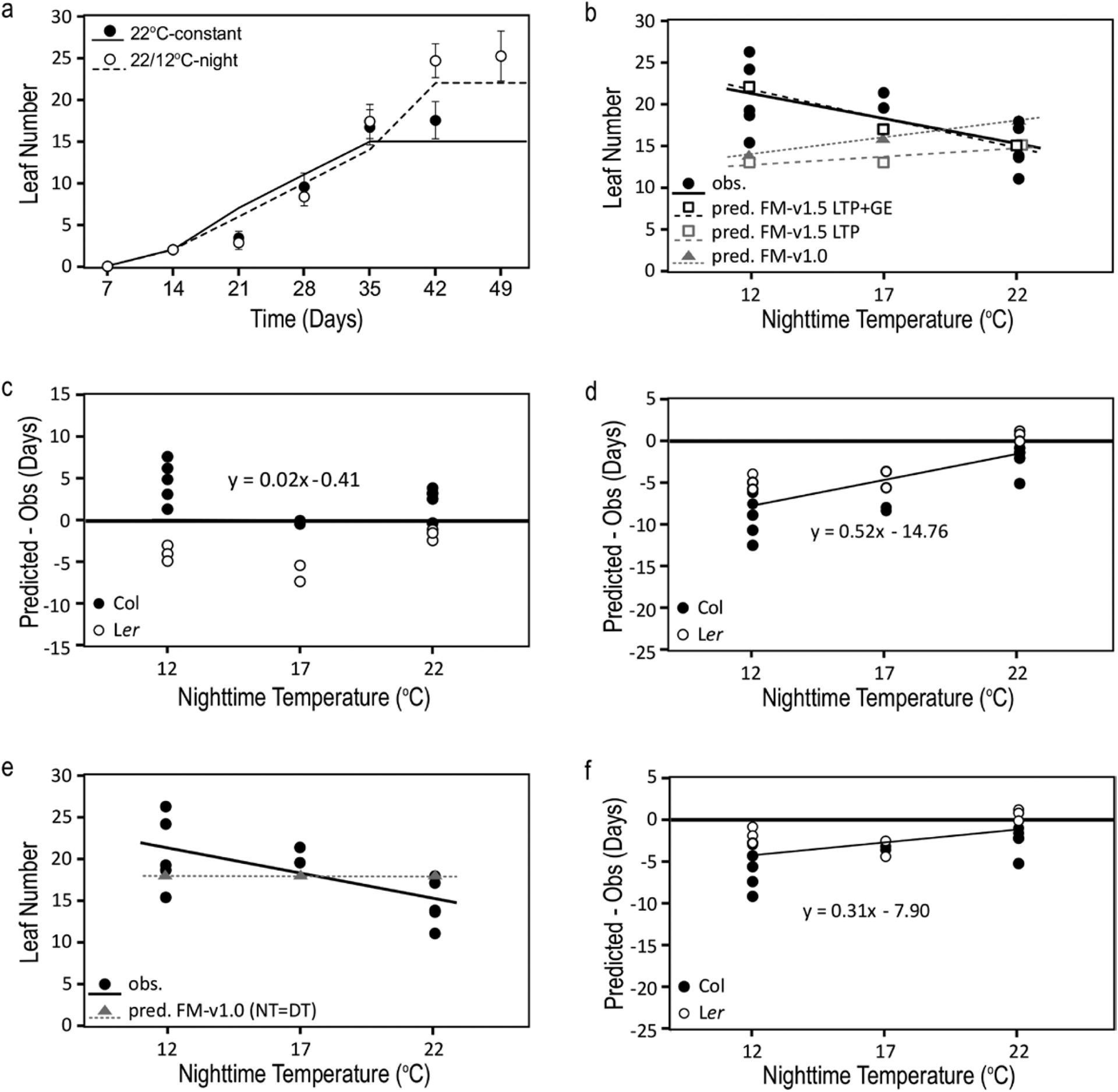
*FT* accumulation as influenced through CO and SVP/FLM and leaf tissue production can improve model predictions in changing temperature conditions compared to MPTUs. (A) Comparison of simulated (lines, FM-v1.5 LTP+GE) and observed (symbols) leaf number by week in Col-0 in constant 22 °C conditions (22 °C-constant) and in 22 °C-day, 12 °C-night temperature cycles (22/12 °C-night). (B) Final leaf number of Col-0 at bolt as observed (obs.) and predicted (pred.) by incorporating temperature influence on *FT* though leaf tissue production (LTP) and *FT* gene expression (GE) (FM-v1.5 LTP+GE), leaf tissue production only (FM-v1.5 LTP), and through traditional MPTUs (FM-v1.0). (C, D) The difference between predicted and observed days to bolt in Col-0 and Landsberg *erecta* (L*er*) using FM-v1.5 LTP+GE (C) and MPTUs in FM-v1.0 (D). (E) Observed and predicted final leaf number and (F) the difference between predicted and observed results using MPTUs in FM-v1.0, adjusted so that daytime and nighttime temperatures are given equal weight. (B–F) Plotted over three nighttime temperatures. Daytime temperature was 22 °C. (C, D, F) Horizontal line at zero is the position in which there is no difference between predicted and observed results. Error bars = 1 SD. If error bars are not visible, the SD is smaller than the height of the symbol. Observed leaf number and days to bolt pooled and compiled from Kinmonth-Schultz *et al*. (2016) (license to reproduce data obtained from John Wiley and Sons, #4601440806514).

We then compared the predicted final leaf number and days to bolt for warm-day, cool-night temperature-cycle treatments in the LTP and LTP+GE model variants in FM-v1.5. Cool temperatures delay bolting and increase leaf number (Blázquez *et al*. 2003; Kinmonth-Schultz *et al*. 2016). In LTP, we expected that cool nighttime temperatures would cause flowering to occur at the same leaf number as the 22 °C-constant control because temperature was not influencing gene expression, but plants would still flower later due to slower whole-plant *FT* accumulation through slower leaf growth. Instead, LTP predicted a leaf number trend opposite to that observed, with a lower leaf number for both 22/17 °C-night and 22/12 °C-night treatments than the 22 °C-constant control (Table 1; Fig. 5B). This behaviour was because leaves that are present in our model continued to produce *FT* such that it continued to accumulate over time as well as with leaf growth. Because leaf production in cooler temperatures was slower, this caused *FT* to reach the threshold at a leaf number lower than the control, rather than the same as the control as we had initially expected. However, as expected, both 22/17 °C-night and 22/12 °C-night treatments bolted later than the 22 °C-constant control (Table 1). The full LTP+GE variant followed a trend close to what we had observed experimentally, increasing the final leaf number for both cool-night temperature treatments and causing a stronger delay in days to bolt than LTP (Table 1; Fig. 5A–C).

We compared this behaviour to flowering predicted using MPTU accumulation by FM-v1.0, adjusting the MPTU threshold to our LD 22 °C-constant conditions, as is recommended when using FM-v1.0 (Chew *et al*. 2014). If FM-v1.0 adequately captured temperature influence, then the MPTU threshold should be similar across treatments, with negligible differences between predicted and observed results for all three temperature regimes. FM-v1.0 predicted fewer leaves in both 22/12 °C-night and 22/17 °C-night conditions than in the 22 °C-constant control, because it reached the MPTU target before reaching the observed final leaf number (Table 1; Fig. 5B) in a manner similar to the behaviour of the FM-v1.5 LTP variant described above. FM-v1.0 accurately captured days to bolt for Col-0 and L*er* grown in 22 °C-constant conditions and showed an expected delay in days to bolt for both 22/12 °C-night and 22/17 °C-night. However, the delay before bolting was shorter than observed (Table 1; Fig. 5D). Recalibrating to equalize the influence of nighttime and daytime temperatures (daytime temperatures are given more weight in FM-v1.0 (Chew *et al*. 2012)) reduced but did not eliminate these trends (Tables 1 and 2; Fig. 5E and F). Therefore, *FT* accumulation can be used to predict flowering and incorporating temperature influence on *FT* transcriptional control improved model predictions in warm-day, cool-night conditions (Table 2).

### Ability of *FT* accumulation to predict flowering after long-term exposure to cool temperature

**Figure 6.**
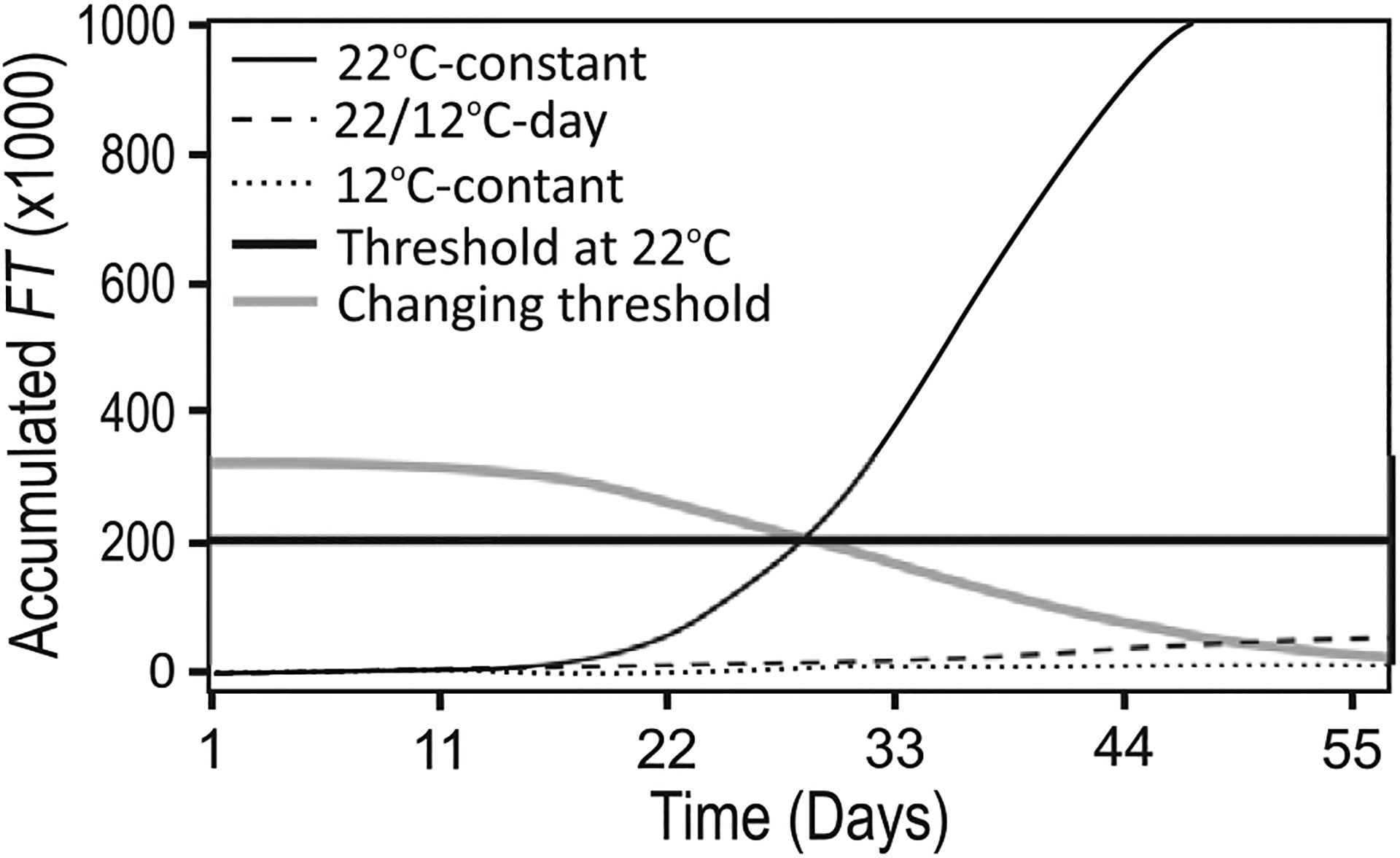
*FT* fails to accumulate to a threshold in some cool-temperature conditions. Plants grown at constant cool (12 °C) temperatures from seed (constant) or after 1 week at 22 °C (22/12 °C-day) do not accumulate *FT* to a threshold set using 22 °C constant temperatures in long days (thick black line). Altering the threshold to decline with developmental time (thick grey line) improves the predictive capacity of FM-v1.5, as we propose in the discussion.

As later produced leaves may lose the capacity to express *FT* (Fig. 3), we wondered how this would impact *FT* accumulation and flowering over longer developmental time periods, such as in cool constant temperatures when *FT* is suppressed and *Arabidopsis* flowers at a higher leaf number (Blázquez *et al*. 2003). We grew Col-0 at 12 °C-constant from seed or in 22/12 °C-day conditions. In the latter treatment, plants germinated and grew for 1 week at 22 °C then remained at 12 °C. Although a boost in *FT* through *CO* is observed in the short term in these conditions, the effect disappears with time [see Supporting Information—Fig. S3b]. We observed flowering at 24 and 28 leaves, respectively, and at 60 and 61 days after plants were moved from cold stratification to growth temperature conditions. In the full FM-v1.5 LTP+GE variant, *FT* failed to accumulate to the threshold set in 22 °C-constant conditions (Fig. 6). After removing the effect of leaf age from the LTP+GE model variant (i.e. equation (3.1) became*FT*_*leaf*_ = *LA* · *FT*, all other components remained the same), *FT* attained the threshold in 22/12 °C-day conditions, but not in 12 °C-constant conditions [see Supporting Information—Fig. S10]. Therefore, whole-plant *FT* accumulation, as influenced by leaf age, leaf tissue production and transcriptional regulation of *FT* by temperature may not be sufficient to predict flowering in conditions in which *FT* is strongly suppressed under the assumption of a constant *FT* threshold.

### Influence of short-term drops in temperature on flowering through *FT* accumulation

Although long-term exposure to cool temperatures suppressed whole-plant *FT* and delayed flowering, rapid temperature drops at dawn in LDs (22/12 °C-day or 22/17 °C-day) caused short-term *FT* induction, even though the temperature then remained cool (Kinmonth-Schultz *et al*. 2016). As *FT* transcript must accumulate over several days before flowering can occur (Krzymuski *et al*. 2015), we wondered whether a rapid temperature drop, causing *FT* induction, could complement *FT* produced in subsequent warm temperatures to accelerate flowering, or if slower whole-plant *FT* accumulation with slower leaf growth, caused by the time in cool temperatures, would delay flowering. To compare the predicted influence of *FT* induction by temperature drops, we used the FM-v1.5 LTP+GE and LTP variants to simulate 2-week-old plants moved to 12 °C in LDs for 2, 4 or 6 days (12 °C-2d, -4d or -6d), then moved to warm, LD conditions. We also grew plants in these conditions. Control plants were moved directly to warm, LD conditions at 2 weeks.

Simulating these conditions in the full LTP+GE variant of FM-v1.5, we found little difference in days to bolt between 12 °C-2d and the control and a 3-day difference between 12 °C-6d and the control. There was a decline in leaf number from 15 leaves in the control to 14 leaves in plants exposed to 12 °C-2d and 12 °C-4d, indicating flowering at a slightly younger developmental age that translated to little difference in days to bolt between the control and 12 °C-2d. In 12 °C-6d, the leaf number increased again to be like the control. In the LTP variant, the leaf number of all three treatments was the same as the control, whereas there was an increase in days to bolt for each consecutive 2 days at 12 °C, consistent with slowed accumulation of *FT* due to slower leaf growth.

**Figure 7.**
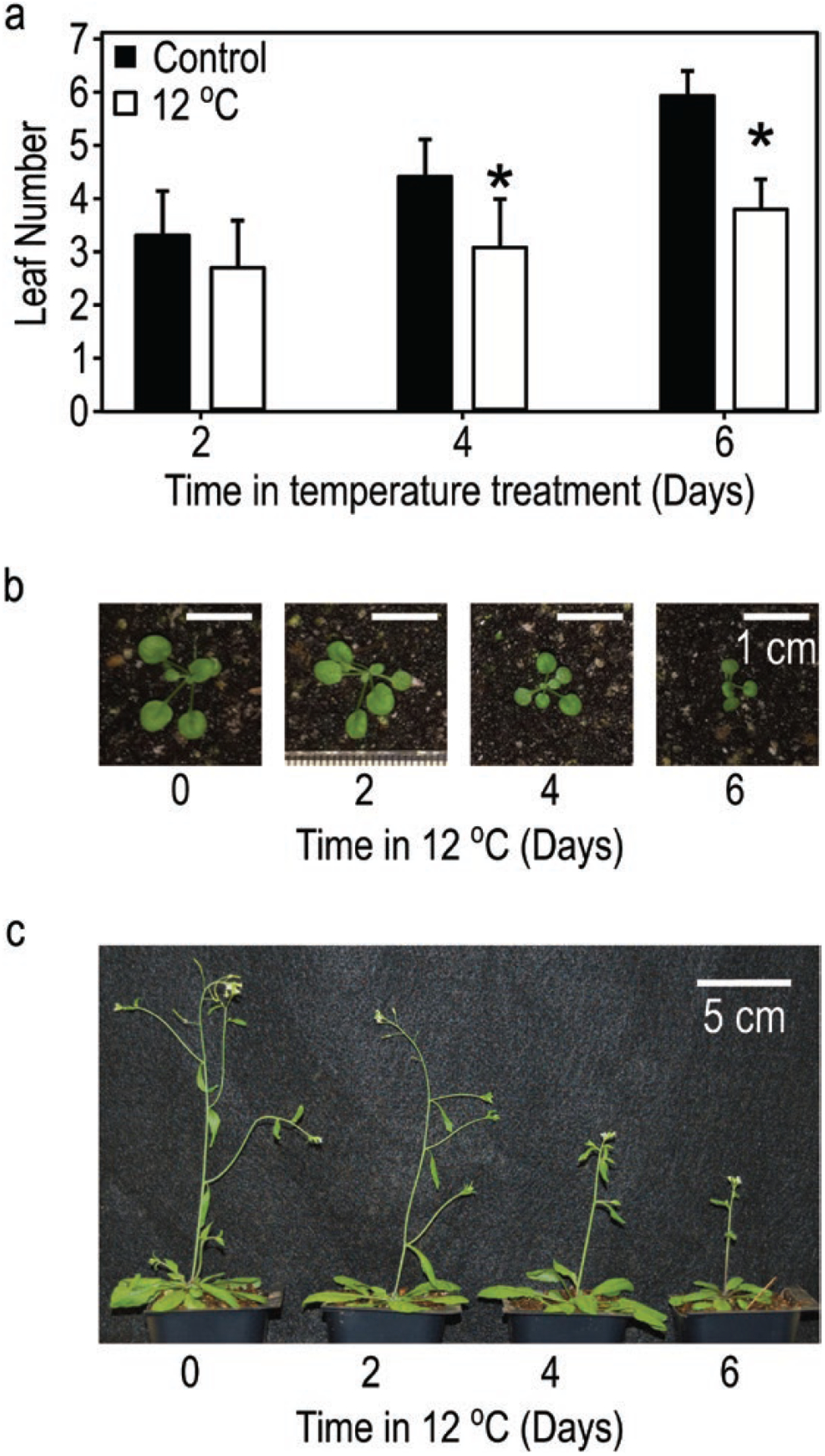
Growth is slowed and flowering is delayed in plants exposed to 12 °C for 2, 4 or 6 days, then returned to warm temperatures (24 °C), relative to control plants grown continuously in warm temperatures. (A) Average leaf number of plants recorded at dawn after 2, 4 or 6 days in 24 °C (control) or 12 °C temperature conditions. (B) Relative seedling sizes on dawn of Day 7, after completion of all cool-temperature treatments (scale bars = 1 cm, 0 = control). Individual images cropped from the same photograph and scaled together (see original image, Supporting Information—Fig. S11). (C) Relative flowering progression 3 days after appearance of last floral stem (bolt) in plants exposed to 12 °C for 2, 4 or 6 days relative to 24 °C control (0, scale bar = 5 cm).

We observed slowed growth (relative to the control) in the cool-temperature treatments. Visible leaf number was significantly lower after 4 and 6 days in 12 °C (Fig. 7A). On Day 7, after completion of all cool-temperature treatments, there was a gradient in leaf area across treatments, with plants from 12 °C-6d being the smallest (Fig. 7B; see Supporting Information—Fig. S11). We observed a statistically significant delay in the number of days to visible bolt in both 12 °C-4d and 12 °C-6d, like both simulations (*P* < 0.001, Table 3; Fig. 7C). While we did not observe a significant difference in leaf number in either 12 °C-2d or 12 °C-4d relative to the control, plants in 12 °C-6d produced approximately 1.5 more leaves before flowering than the other three treatments (*P* < 0.001), more like the predicted increase in leaf number from 12 °C-2d and 12 °C-4d to 12 °C-6d in the LTP+GE model variant (Table 3). Combining both the simulated and observed results, it appears that the boost in *FT* that occurred in 12 °C-2d or 12 °C-4d conditions seems to have compensated for the slightly slower whole-plant *FT* accumulation in those conditions, causing plants in those conditions to bolt at a similar developmental stage (leaf number) as the control. However, because growth was slowed in those conditions, plants in 12 °C-4d bolted later in terms of number of days. In the 12 °C-6d conditions, the early boost in *FT* faded over time and was not enough to compensate for the overall slower whole-plant *FT* accumulation due to slowed growth. Therefore, plants in these conditions bolted both at a later developmental stage (higher leaf number) and at a higher number of days.

## Discussion

To explore the mechanisms underlying the long-observed thermal time responses, we set out to address whether accumulation of *FT* as a function of leaf growth as influenced by temperature could be used to predict flowering times in a manner like thermal time. Here, we found that the FM-v1.0 model using thermal time (MPTUs) and our FM-v1.5 LTP variant, in which *FT* accumulated only with leaf growth as influenced by temperature behaved similarly (Table 1; Fig. 5B and D), each predicting delays in days to bolt under temporal shifts in temperature regimes in LDs relative to the constant-temperature control. This indicates that accumulation of the *FT* gene product with leaf tissue production could be a component of thermal time. However, the delays in warm-day, cool-night conditions for each model were less than observed. The second question we addressed was whether incorporating direct temperature influence on *FT* could improve model predictions when there are temporal shifts in temperature regimes. Indeed, adding direct temperature regulation of *FT* improved model predictions by increasing the degree of predicted difference between the warm-day, cool-night treatments and the control. This suggests that incorporating underlying molecular mechanisms into models of plant development could improve model utility for a range of conditions without requiring recalibration (White 2009; Boote *et al*. 2013; Chew *et al*. 2017).

Our work additionally suggests avenues for future study to help us better understand how the external environment acts to influence underlying molecular components to yield developmental phenotypes. We found that *FT* was reduced in later-produced leaves (Fig. 3). This change in *FT* expression with developmental age was incorporated into FM-v1.5 using leaf age as a proxy and caused *FT* to fail to accumulate to a preset threshold to predict flowering in constant cool temperatures. This finding enables integration of qualitative (presence/absence) and quantitative (dosage response) aspects of *FT* effects on flowering and has implications for other conditions in which *FT* is suppressed, such as in short day lengths. It can help us quantify when *FT* plays a role during development, when *FT* alone is a poor predictor of flowering, and when it may act synergistically or competitively with other flowering factors.

For instance, the *FT* threshold requirement should be influenced by shoot-apex genes, and their sensitivity likely changes with climate and developmental age. For example, SVP acts both in the leaves to regulate *FT* and at the shoot apex to regulate *SUPPRESSOR OF OVEREXPRESSION OF CONSTANS* (*SOC1*). Currently, the latter mechanism is not captured in our model. In short days, high temperatures may reduce SVP activity at the shoot apex to initiate flowering despite lower *FT* levels (Fernández *et al*. 2016). At the shoot apex, SVP suppresses *SOC1*, which is positively regulated by *FT*, and which activates *LEAFY* (*LFY*), a key player in the floral transition (Schmid *et al*. 2003; Lee *et al*. 2008; Jang *et al*. 2009). FT protein also activates *APETALA1* (*AP1*) at the shoot apex (Lee and Lee 2010). AP1, in turn, is involved in the down-regulation of *TERMINAL FLOWER 1* (*TFL1*), a *FT* homolog. TFL1 is thought to compete with FT for binding with FD to suppress *LFY* and *AP1*, forming a negative feedback loop (Kaufmann *et al*. 2010; Wickland and Hanzawa 2015). Both the decrease in SVP and TFL1 would likely decrease the *FT* threshold needed to induce flowering. Like *SVP*, *TFL1* may be temperature sensitive (Kim *et al*. 2013).

A changing threshold, due to different *LATE FLOWERING* alleles in Pea, a homologue of *TFL1* in *Arabidopsis* (Foucher *et al*. 2003), aided flowering time predictions (Wenden *et al*. 2009). Incorporating such a mechanism—influenced by climate and developmental age—may aid understanding of how climate influences flowering. As proof of concept, we caused the *FT* threshold level to change with developmental age (thermal time) (Fig. 6). Doing so improved the predictive capacity of FM-v1.5 in constant, cool temperatures, and could be used to incorporate the action of SVP and other players at the shoot apex.

SVP, in conjunction with FLM, suppresses *FT* in response to cool temperatures (Blázquez *et al*. 2003; Lee *et al*. 2007, 2013). We demonstrated that residual SVP and FLM activity after short-term cold exposures could be important for *FT* regulation. For instance, to mimic observed dusk suppression of *FT* in warm-day, cool-night temperature cycles, simulated SVP/FLM activity decayed slowly after at 12 °C night, such that it was higher after 16 h at 22 °C, than it was in constant 22 °C conditions.

Our model also highlights the need to clarify the degree of temperature influence in *FT* activation and suppression at a range of temperatures. For example, in FM-v1.5, *FT* is not induced to observed levels, and induction is not maintained as long, after dawn exposure to 17 °C (Fig. 2E). It is possible that SVP activation is lower in 17 °C, than predicted from our model. Despite this, the relative difference in transcript levels across treatments was similar to the relative difference in daytime *FT* expression, which correlates strongly with flowering (Krzymuski *et al*. 2015; Kinmonth-Schultz *et al*. 2016) enabling FM-v1.5 to be useful in exploring *FT* accumulation and its influence on flowering.

Our simulations, while requiring validation in other temperature conditions, are consistent with approaches that use day length and vernalization to influence the leaf number at which the reproductive transition occurs (Brown *et al*. 2013). However, our work demonstrates that ambient temperature should be incorporated to influence leaf number as well, not only developmental rate. For instance, we altered *FT* accumulation, either by removing temperature influence on *FT* transcription (FM-v1.5 LTP, Fig. 4; Table 2) or by short-term, cool-temperature exposure (Fig. 2A; Table 3), affecting final leaf number. In each instance, *FT* still accumulated with leaf production as influenced by temperature, demonstrating that temperature influences when (in days) the reproductive transition occurs by influencing the developmental rate and whole-plant *FT* accumulation. We further suggest that tissue accumulation through growth is an underlying factor in the accumulation of thermal time as it causes gene products to accumulate. Together, this work demonstrates that decomposing the influences of climate and development can improve our understanding of plant responses in a range of conditions.

## Supplementary Material

1 **Appendix S1**. Equation used in FM-v1.0 to describe *FT* transcription as a function of CO protein (Chew *et al*. 2014).
**Table S1**. Coefficients and values for equations used in FM-v1.5.
**Figure S1**. Graphic representation of FM-v1.0.
**Figure S2**. SVP/FLM activity declines over time.
**Figure S3**. Behavior of *CO* mRNA in response to different temperature regimes.
**Figure S4**. Simulated expression profile of *LHY*, plotted over time used to increase morning transcriptional sensitivity of *FT*.
**Figure S5**. Simulated *FT* expression profile in FM-v1.5 in the *svp* mutant mimics the pattern but not relative amplitude of that observed.
**Figure S6**. The spatial expression profile of *FT* changes with leaf age.
**Figure S7**. Behaviour of morphological and physiological parameters in FM-v1.0 and v1.5.
**Figure S8**. Specific leaf area (SLA) declines after growth in cool constant temperatures or in warm-day, cool-night temperature cycles relative to a constant, warm-temperature control. (Pyl *et al*. 2012).
**Figure S9**. *FT* expression in plants grown from seed at constant cool temperatures or moved to constant cool temperatures at dawn after 1 week in 22 °C.
**Figure S10**. In cool-temperature conditions, in which *FT* expression is suppressed, *FT* accumulation reaches a threshold set in 22 °C-constant conditions only after the influence of leaf age on capacity of different leaves to express *FT* is removed from the model.
**Figure S11**. Growth is slowed in plants exposed to 12 °C for 2, 4 or 6 days.

## Figures and Tables

**Table 1.** Observed and simulated days to bolt and leaf number in Columbia-0 (Col-0) and Landsberg *erecta* (L*er*) plants exposed to drops in nighttime temperature.

Strain		Treatment	Obs. data	FM-v1.0	FM-v1.5 LTP+GE	FM-v1.5 LTP
Col-0	Days to bolt	22 °C day 22 °C night	32.27	30.33	35.00	35.00
		22 °C day 17 °C night	38.60	30.63	38.50	35.96
		22 °C day 12 °C night	40.08	31.25	44.88	37.50
	Leaf number	22 °C day 22 °C night	14.77	18.00	15.00	15.00
		22 °C day 17 °C night	20.50	16.00	17.00	13.00
		22 °C day 12 °C night	20.79	14.00	22.00	13.00
Ler	Days to bolt	22 °C day 22 °C night	27.13	27.75	25.42	25.42
		22 °C day 17 °C night	32.75	28.29	26.50	25.67
		22 °C day 12 °C night	33.23	28.63	29.33	25.79
	Leaf numbers	22 °C day 22 °C night	7.40	14.00	8.00	8.00
		22 °C day 17 °C night	8.17	13.00	8.00	7.00
		22 °C day 12 °C night	9.98	12.00	9.00	7.00

**Table 2.** Fit of FM-v1.0 and FM-v1.5 for Col-0 and L*er* combined.

		FM-v1.0	FM-v1.0 (Night = Day)	FM-v1.5 LTP+GE
Days to bolt	RMSE	5.65	3.69	3.95
	Bias	−4.30	−2.69	0.11
Leaf number	RMSE	5.56	4.91	2.67
	Bias	0.79	2.33	−0.07

**Table 3.** Flowering times in plants shifted from warm (24 °C) temperature conditions to, and remaining in, cool-temperature conditions (12 °C) for 2, 4 or 6 days.

	Treatment	Obs. data	*n*	Robust SE	Robust Z	*P*-value^a^	CI of dif. (lower)^a^	CI of dif. (upper)^a^	FM-v1.5 LTP+GE	FM-v1.5 LTP
Days to bolt	LD24C (int)	35.00	11	0.62	31.10				34.75	34.75
	12C, 2d	36.00	15	0.60	1.73	0.08	−0.65	2.73	34.92	35.75
	12C, 4d	37.31	14	0.42	5.54	0.00	0.87	3.80	35.83	36.62
	12C, 6d	38.64	14	0.35	7.85	0.00	2.24	5.02	37.79	37.58
Leaf number	LD24C (int)	13.73	11	0.52	19.59				15.00	15.00
	12C, 2d	13.13	15	0.34	−1.21	0.23	−0.81	1.65	14.00	15.00
	12C, 4d	13.64	14	0.36	0.49	0.63	−1.07	1.42	14.00	15.00
	12C, 6d	15.14	14	0.39	3.47	0.00	0.08	2.64	15.00	15.00

Observed and simulated days to bolt and leaf number of rosette leaves on the main stem in Columbia plants exposed to short-term drops to 12 °C temperature relative to plants remaining in the warm temperature control (24 °C) in long days (LD).

a Observed treatments counted significantly different from the control when *P* < 0.05 and the CI of the difference from the control does not contain zero, both were lowered from these values using the Bonferonni correction to account for multiple comparisons.
