# Supplementary material for: An explanatory model of temperature influence on flowering through whole-plant accumulation of FLOWERING LOCUS T in Arabidopsis thaliana
Source: In Silico Plants. Author manuscript; Available in PMC 2022 Oct 5. (PMC9534314; doi:10.1093/insilicoplants/diz006)

S1 Appendix: Equations

In FM-v1.0, *FT* tracks CO protein levels as below.

(S1.1)
$$\frac{dFT}{dt} = B_{CO} \cdot \left(V_{CO_1} \frac{CO_p}{K_{CO_1} + CO_p} - v_{FT_1} \frac{FT}{k_{FT_1} + FT} \right)$$

Reference

Chew YH, Wenden B, Flis A, Mengin V, Taylor J, Davey CL, et al. Multiscale digital *Arabidopsis* predicts individual organ and whole-organism growth. PNAS. 2014;111:E4127–36.

S1 Table: Parameters and variables for equations used in FM-v1.5.

Eq.	Parm.	Description	Value	Units
1.1	$SVP_{p\text{-}new}$	The rate of SVP protein production	-	nmol h^{-1}
1.1	T	Tissue temperature assumed to be equal to air temperature	-	˚C
1.1	b_1	The SVP production extrapolated to T= 0 ˚C	4.50	nmol h^{-1}
1.1	b_2	The degree of reduction of SVP production with temperature	0.25	˚C^{-1}
1.2	SVP_{ceil}	The ceiling of the SVP production at day d_{FTL}	-	nmol h^{-1}
1.2	d_{FTL}	Days after emergence of first true leaves	-	d
1.2	b_3	The rate of decline in SVP_{ceil} over d_{FTL}	0.20	d^{-1}
1.2	SVP_0	SVP production before true leaf emergence	7.3891	nmol h^{-1}
1.3	SVP_p	Relative amount of SVP protein at a given time		nmol
1.3	v_{SVP}	Rate constant for degradation of SVP protein	0.05	h^{-1}
1.4	K_T	Temperature influence on *CO* mRNA induction	-	-
1.4	c_1	The change in *CO* mRNA production with the difference between the maximum and current temperatures	0.2735	˚C^{-1}
1.4	c_2	Change in relative induction of *CO* over time	0.2731	d^{-1}
1.5	$CO_{m\text{-}new}$	*CO* mRNA as influenced both by the circadian clock and temperature changes	-	nmol
1.5	CO_m	*CO* mRNA as generated based on the circadian clock, it is an output of the original photoperiodism module in FM-v1.0	-	nmol
1.5	CO_p	Relative amount of CO protein	-	nmol
1.6	v_{CO_m}	Rate constant of *CO* mRNA translation	7	h^{-1}
1.6	v_{CO_p}	Maximum rate of ligh-dependent CO protein degradation	60	nmol h^{-1}
1.6	k_{CO_p}	Concentration of CO protein at which the decay rate is ½ v_{CO_p}	2	nmol
1.6	L_1	0 at night, 1 during the day. Ensures CO protein degradation occurs only at night. The value is continuous but changes rapidly between 0 and 1 at dawn and dusk	1 or 0	-
1.7	FT_m	Relative amount of *FT* mRNA as	-	nmol

		influenced by temperature at the site of transcription		
1.7	L_2	Switch that boosts *FT* transcriptional sensitivity in the morning, artificially uses *LHY* from the clock component of photoperiodism module	1 or 1.3	-
1.7	V_{CO}	Maximum rate of *FT* mRNA synthesis with CO protein	5.11	nmol h^{-1}
1.7	K_{CO}	The concentration of CO protein at which the rate of *FT* synthesis is ½ V_{CO}	17.50	nmol
1.7	K_{SVP}	The equilibrium constant describing the strength with which SVP/FLM binds to *FT* regulatory regions	0.25	nmol
1.7	v_{FT}	Maximum rate of *FT* mRNA decay	14.50	nmol h^{-1}
1.7	k_{FT}	The concentration of *FT* at which the rate of decay is ½ v_{FT}	14.50	nmol
2.1	β_{FT}	"capacity" of each leaf to express *FT* based on age	-	-
2.1	β_{FTmax}	Scaling factor influencing the maximum capacity for *FT* expression of leaves on a plant – dependent on plant age (i.e. total number of leaves) (eq. 2.2)	-	-
2.1	r	Relative leaf age, with the newest leaf being 1	-	-
2.1	R_{crit}	Relative leaf age for the oldest leaf capable of expressing *FT*	30	-
2.1	R_{opt}	Relative age at which maximum β_{FT} is attained – dependent on number of leaves per plant (eq. 2.3)	-	-
2.1	e	Describes the curvature of the slope as *FT* increases and decreases with leaf age	12	
2.2	g_1	Term relating the maximum level of *FT* expression to total leaf number	2.25	
2.2	l	Current total number of leaves	-	-
2.3	g_2	Term relating total leaf number to the leaf (based on relative leaf age) that expresses the highest level of *FT*	0.65	
3.1	FT_{leaf}	Relative *FT* per leaf per leaf area	-	nmol cm^{-2}
3.1	LA	Total leaf area of each leaf, an output of the Functional Structural Plant module	-	cm^2
3.1	FT_{plant}	Relative total *FT* mRNA amount of a whole-plant	-	nmol $plant^{-1}$

3.1	FT_{thresh}	In the model, *FT* accumulates to this threshold in a manner like thermal time accumulation. Values shown are for a threshold that is constant through developmental time and are genotype specific	Col-0 207704.17<br><br>L*er* 21436.43	nmol plant^{-1}
S1.1	B_{CO}	CO-independent transcription rate [1]	0	nmol h^{-1}
S1.1	V_{CO_1}	Maximum rate of *FT* mRNA synthesis with CO protein [2]	0.58	nmol h^{-1}
S1.1	K_{CO_1}	The concentration of CO protein at which the rate of *FT* synthesis is ½ V_{CO_1} [2]	7.3533	nmol
S1.1	v_{FT_1}	Maximum rate of *FT* mRNA decay [2]	1.8674	nmol h^{-1}
S1.1	k_{FT_1}	The concentration of *FT* at which the rate of decay is ½ v_{FT_1} [2]	5.3925	nmol

References

1. Salazar JD, Saithong T, Brown PE, Foreman J, Locke JCW, Halliday KJ, et al. Prediction of photoperiodic regulators from quantitative gene circuit models. Cell. 2009;139:1170–9.

2. Chew YH, Wenden B, Flis A, Mengin V, Taylor J, Davey CL, et al. Multiscale digital *Arabidopsis* predicts individual organ and whole-organism growth. PNAS. 2014;111:E4127–36.

S1 Fig. Graphic representation of FM-v1.0

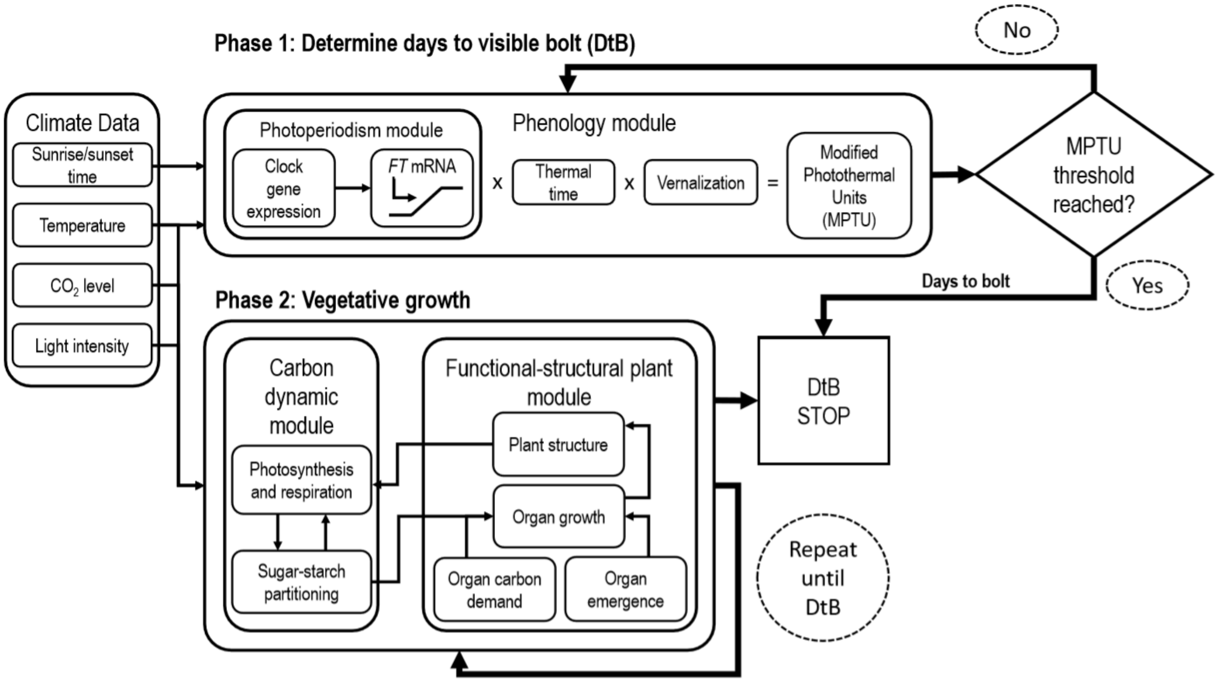

Reference

Chew YH, Wenden B, Flis A, Mengin V, Taylor J, Davey CL, et al. Multiscale digital *Arabidopsis* predicts individual organ and whole-organism growth. PNAS. 2014;111:E4127–36.

S2 Fig. SVP/FLM activity declines in influence of *FT* over time. **(a-b)** The ratios of actual **(a,** Kinmonth-Schultz *et al.* 2016) and simulated **(b)** peak *FT* levels 16-hr after dawn in long days (LD) in 22˚C-day,12˚C-night (22-12˚C) temperature cycle conditions to 22˚C constant temperature conditions. Dotted line = Best-fit line. The slope is calculated beginning at day 2 **(a)** and day 4 **(b)** when *FT* suppression in cool-night conditions is at the maximum. **(c)** Simulated SVP/FLM activity over two weeks in the following conditions: 22˚C constant (22˚C-constant), 22˚C-day 12˚C-night temperature cycles (22/12˚C-night), and 22˚C constant shifted to 12˚C constant at dawn (22/12˚C-day). Light bars = day, black bars = night. License to reproduce data contained in Kinmonth-Schultz *et al.* 2016 obtained from John Wiley and Sons, #4601440806514.

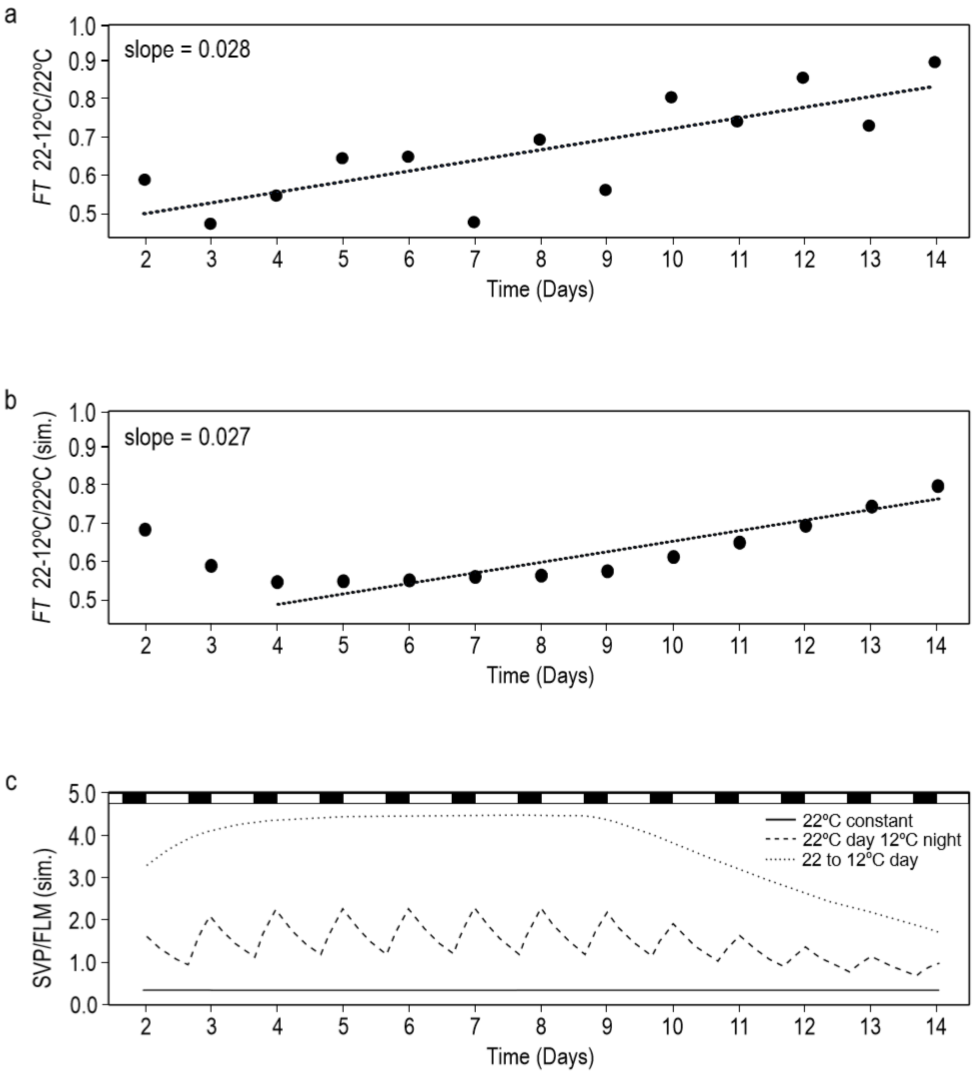

Reference

Kinmonth-Schultz HA, Tong X, Lee J, Song YH, Ito S, Kim S-H, et al. Cool night-time temperatures induce the expression of *CONSTANS* and *FLOWERING LOCUS T* to regulate flowering in *Arabidopsis*. New Phytol. 2016;211:208–24.

S3 Fig. Behavior of *CO* mRNA in response to different temperature regimes. **(a)** The ratio of *CO* mRNA at its peak to *CO* mRNA at dusk in treatments in which the daytime temperature was 22˚C and the nighttime temperature was 22˚C**,** 17˚C, or 12˚C (0, 5, or 10 ˚C difference). These values are relativized to the 22˚C control in each experiment. **(b)** The levels of *CO* transcript after a drop from 22 to 12˚C relative to levels in plants that had remained at 22˚C. The drop from 22 to 12˚C occurred just after dawn on day 1. The daily averages of three time points (ZT0, 8, and 16) were compared.

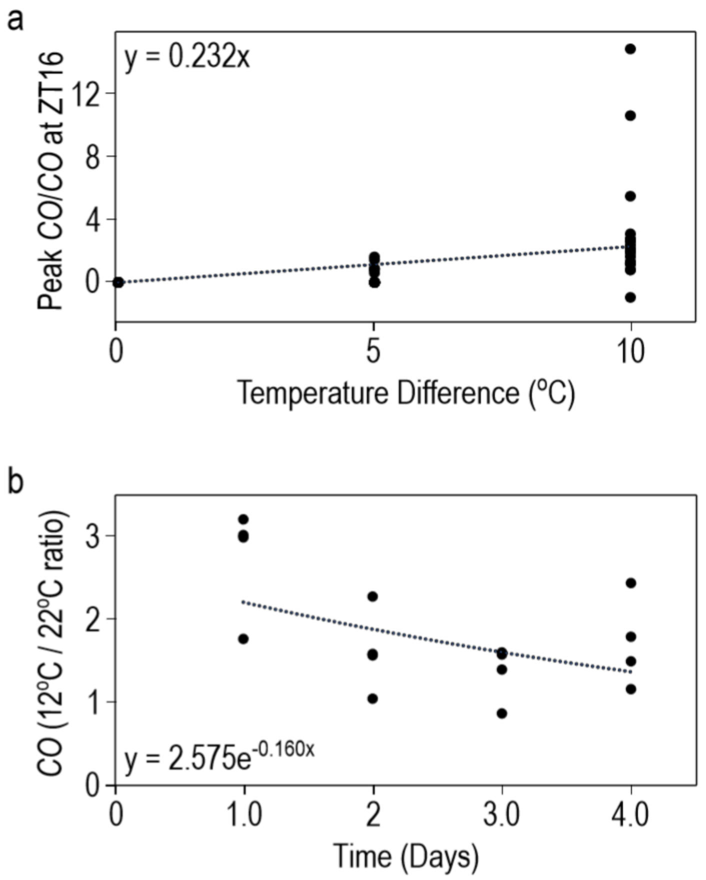

S4 Fig. Simulated expression profile of *LHY*, plotted over time (solid line). Morning sensitivity of FT is simply a switch. When *LHY* exceeds 0.4 (dotted line), then L_2 in equation [1.7] is 1.3, otherwise it is 1.

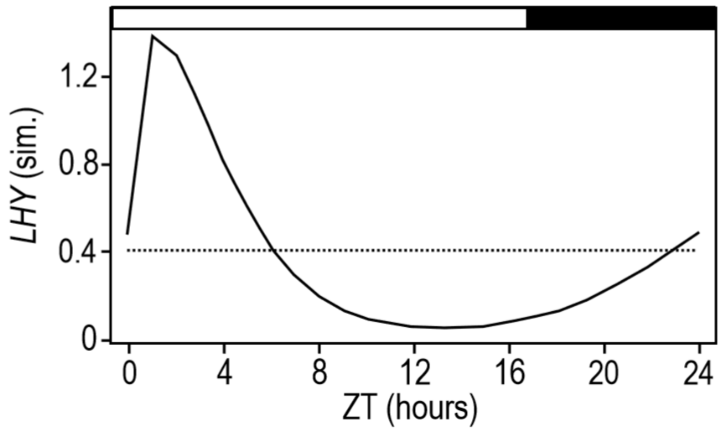

S5 Fig. Simulated *FT* expression profile in FM-v1.5 in the *svp* mutant mimics the pattern but not relative amplitude of that observed. Observed pattern of *FT* in an *svp* mutant **(a**, Kinmonth-Schultz *et al*. 2016**)** and the simulated pattern of *FT* when the term for SVP/FLM activity is set to zero **(b)** in warm (22˚C)-day, cool (12˚C)-night temperature-cycle treatments or in 22˚C constant temperature control. The white and black bars represent light and dark periods, respectively. License to reproduce data contained in Kinmonth-Schultz *et al.* 2016 obtained from John Wiley and Sons, #4601440806514.

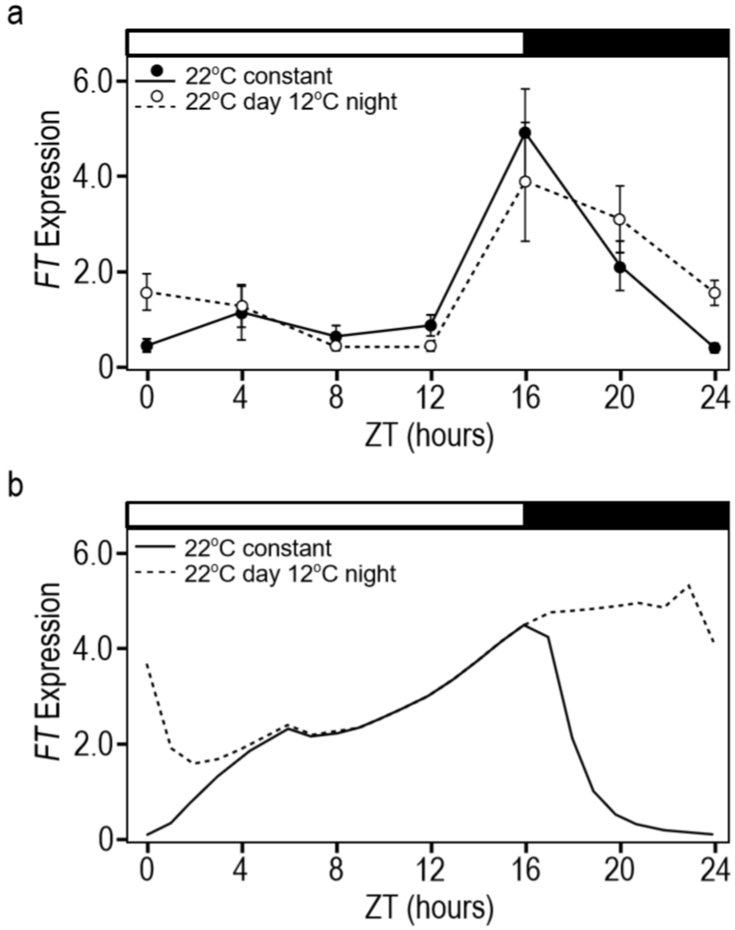

Reference

Kinmonth-Schultz HA, Tong X, Lee J, Song YH, Ito S, Kim S-H, et al. Cool night-time temperatures induce the expression of *CONSTANS* and *FLOWERING LOCUS T* to regulate flowering in *Arabidopsis*. New Phytol. 2016;211:208–24.

S6 Fig. The spatial expression profile of *FT* changes with leaf age. The staining pattern of *pFT:GUS* plants aged two, three, four, and six weeks 16 hours after dawn after exposure to long days or short days for three days. Images were compiled from smaller microscope images as leaves were too large for a single frame. The traced images were used to determine percent leaf area stained, the un-traced images are shown for comparison. Leaves are ordered by relative leaf age, with one being the first to emerge. Leaves shown are from representative plants of each age. Scale bars = 2.5 mm unless indicated by an asterisk (* = 1 mm, ** = 0.5 mm).

(a) Long Days (Traced)

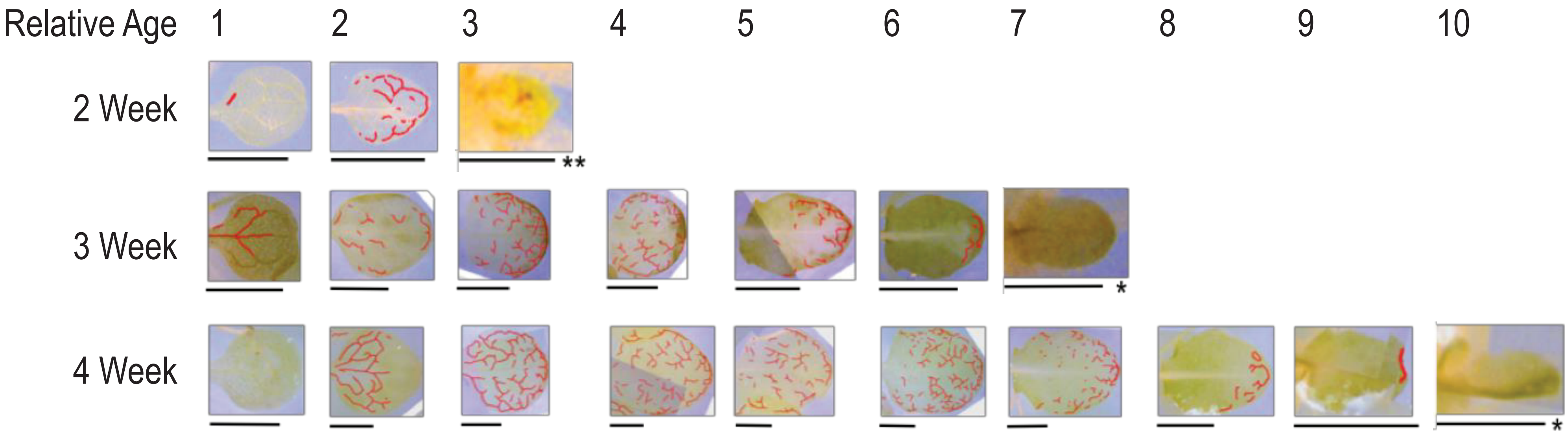

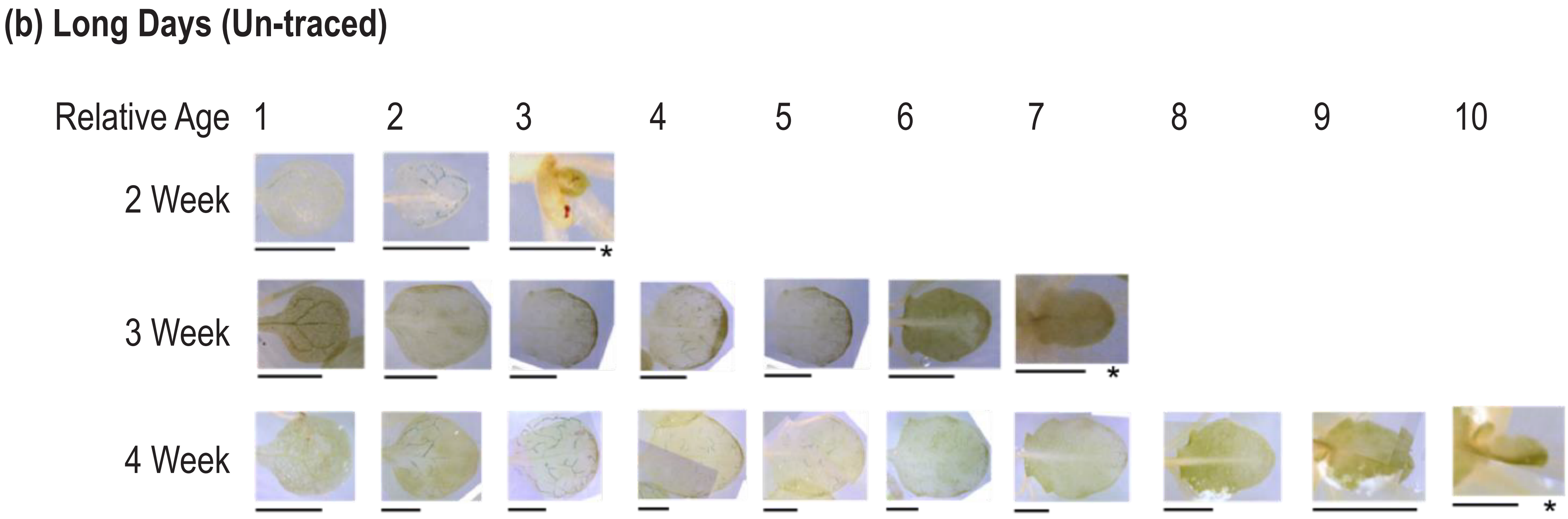
(b) Long Days (Un-traced)
Relative Age
1
2
3
4
5
6
7
8
9
10
2 Week
3 Week
4 Week
*
*
*

(c) Short Days (Traced)

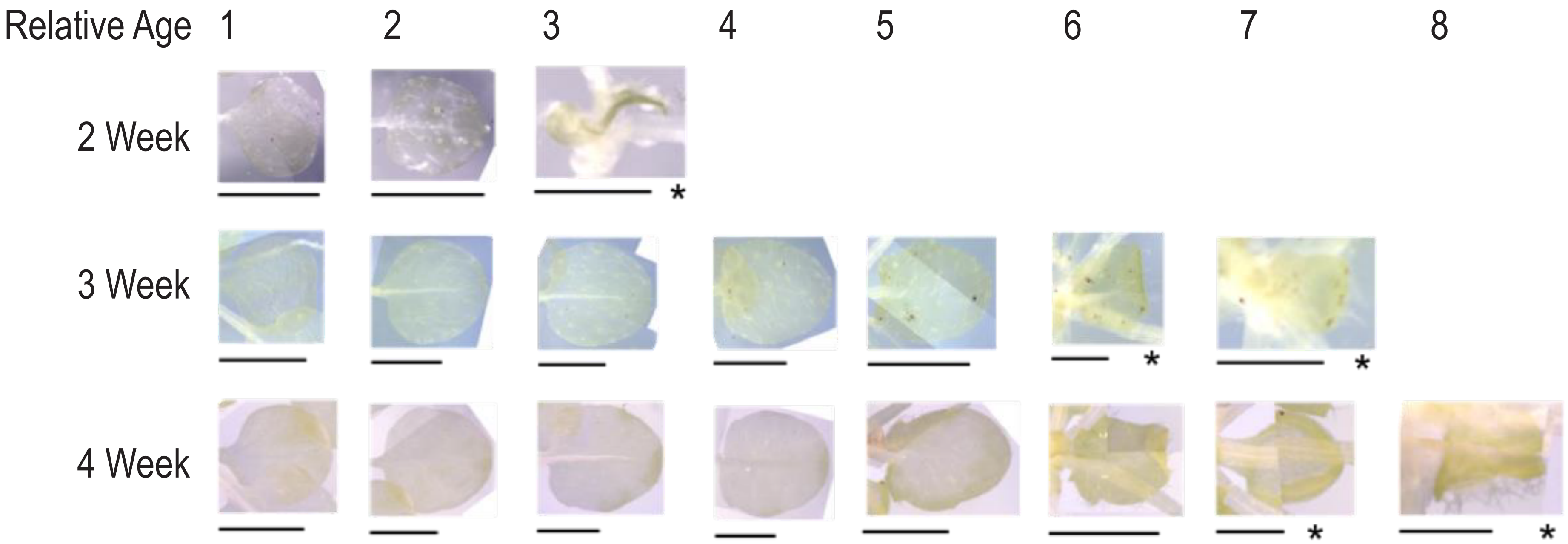

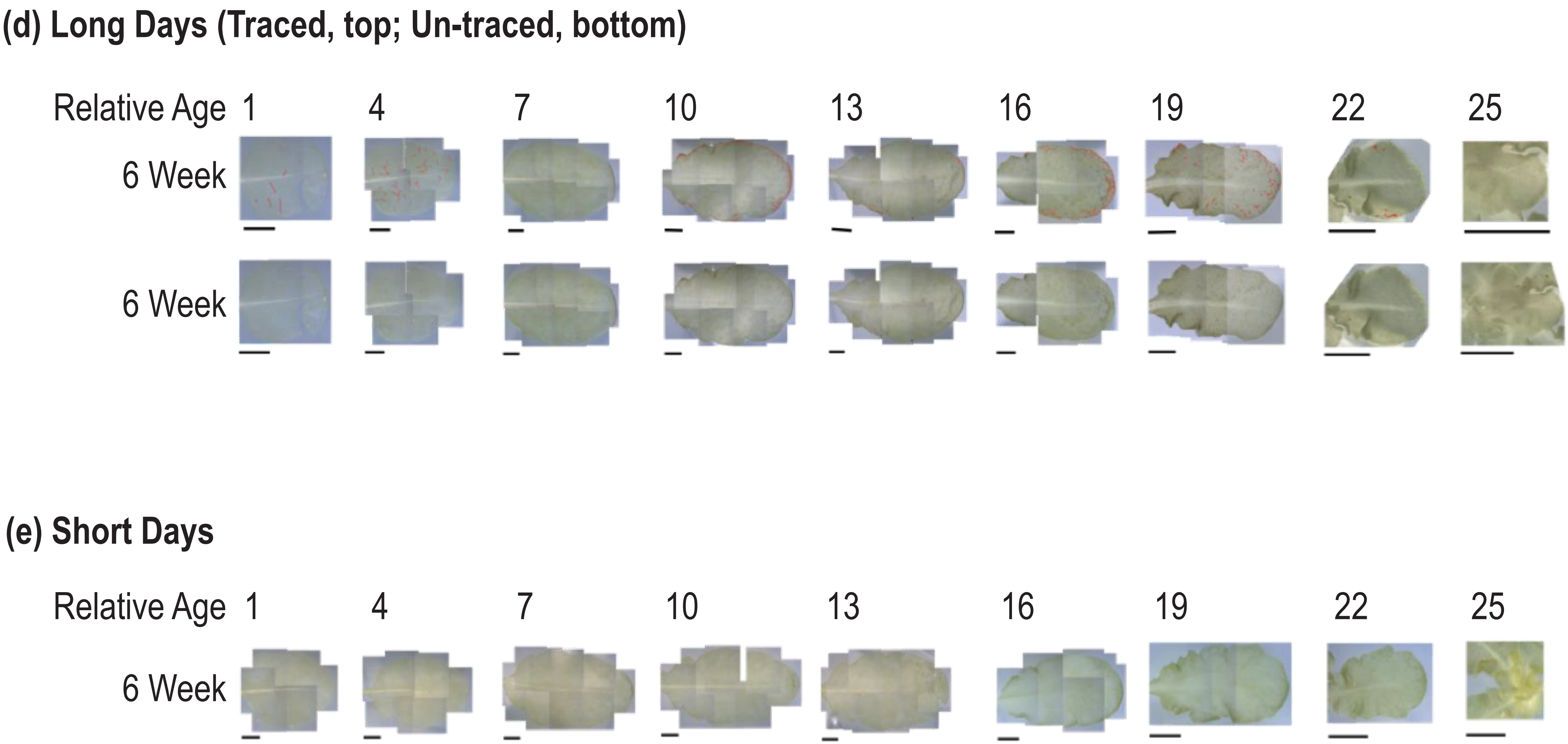
(d) Long Days (Traced, top; Un-traced, bottom)
Relative Age 1 4 7 10 13 16 19 22 25
6 Week
6 Week
(e) Short Days
Relative Age 1 4 7 10 13 16 19 22 25
6 Week

S7 Fig. Behavior of morphological and physiological parameters in FM-v1.0 **(a-e)** and v1.5 **(f)**. **(a)** Leaf area is higher in cool-night temperature conditions (dashed lines), in both long days (LD, black lines, light intensity = 60 µmol m^2 s^{-1}) and short days (SD, green lines, light intensity = 140 µmol m^2 s^{-1}). Leaf area in FM-v1.0 and 1.5 responds to light intensity and is larger in SDs because of higher light intensity. **(b, c)** Specific leaf area (SLA **(b)**) declines with increasing developmental time (thermal time **(c)**), and therefore declines more slowly in cool-night temperature conditions (lines for SD and LD 22˚C constant temperature conditions overlap). **(d)** Net CO_2 assimilation does not differ between 22˚C constant (22˚C-constant) and 22˚C day, 12˚C night temperature-cycle (22/12˚C-night) conditions (SD shown, lines overlap completely). **(e)** Maintenance respiration slows at night in 22/12˚C-night temperature-cycle conditions relative to 22˚C-constant, rising briefly at dawn when the temperature increases (peak, dashed line, SD shown). **(f)** Modification in FM-v1.5 cause SLA to be lower in cool-temperature conditions).

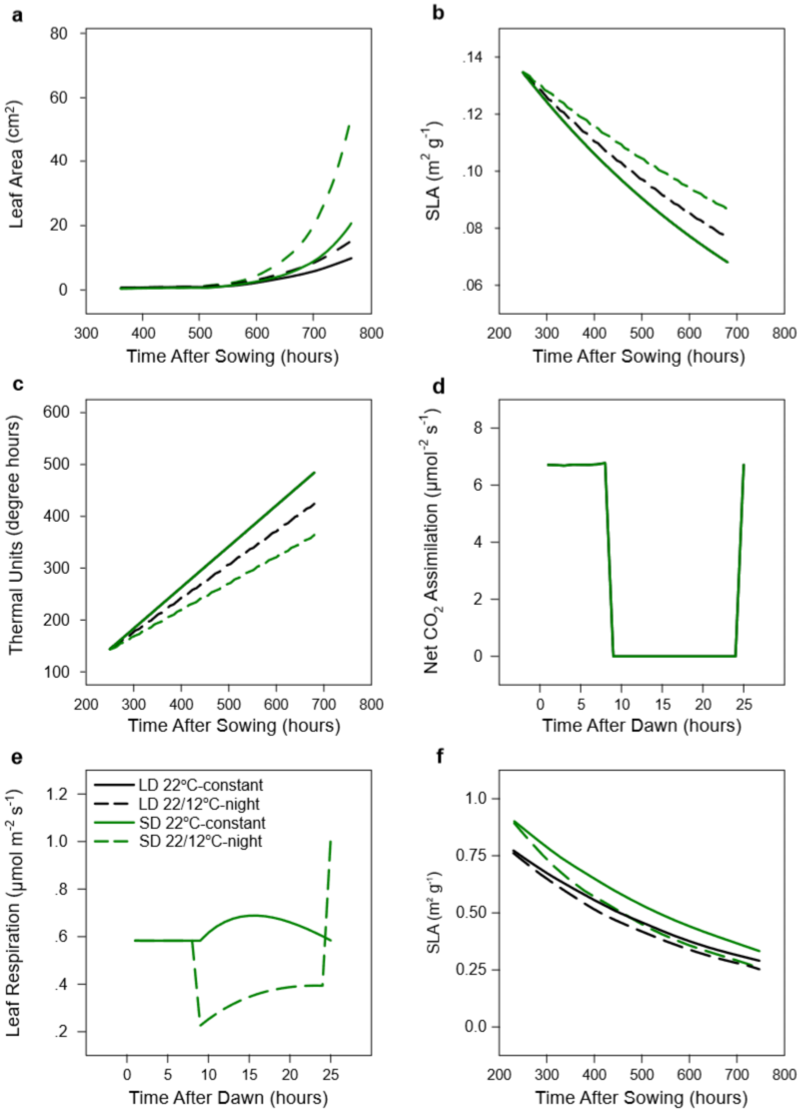

S8 Fig. Specific Leaf Area (SLA) declines after growth in cool constant temperatures or in warm-day, cool-night temperature cycles (24/16 or 12˚C-night) relative to a constant, warm-temperature control (24˚C). Data on the *Arabidopsis* Columbia strain extrapolated from Pyl *et al*. 2012. License to reproduce data contained in Pyl *et al.* 2012 published by American Society of Plant Physiologists, #4601451256232.

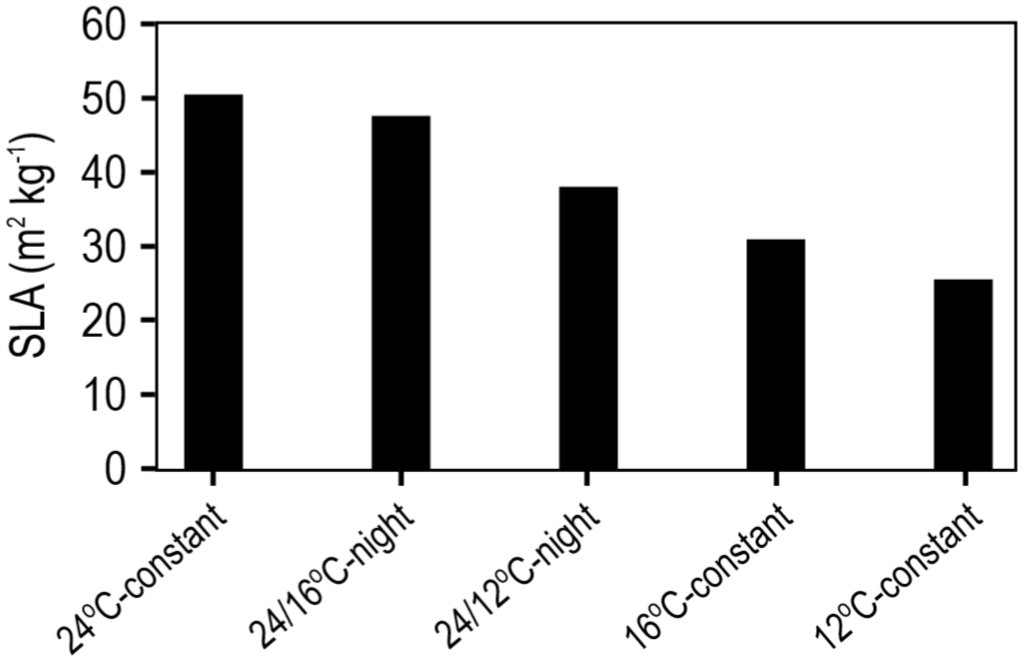

Reference

Pyl E-T, Piques M, Ivakov A, Schulze W, Ishihara H, Stitt M, et al. Metabolism and growth in *Arabidopsis* depend on the daytime temperature but are temperature-compensated against cool nights. Plant Cell. 2012;24:2443–69.

S9 Figure. Observed diurnal patterns of *FT* gene expression in constant-temperature conditions (22, 17 and 12˚C) and in conditions in which the temperature dropped from 22˚C to 17 or 12˚C at dawn, then remained at the cooler temperature (22/12 or 17˚C-day) relative to the 22˚C-constant temperature control (Kinmonth-Schultz *et al.* 2016). The x-axis is in zeitgeber time (ZT) and represents hours after dawn. The white and black bars represent light and dark periods respectively. Error bars = 1 S. E. If error bars are not visible, the S. E. is smaller than the height of the symbol. License to reproduce data contained in Kinmonth-Schultz *et al.* 2016 obtained from John Wiley and Sons, #4601440806514.

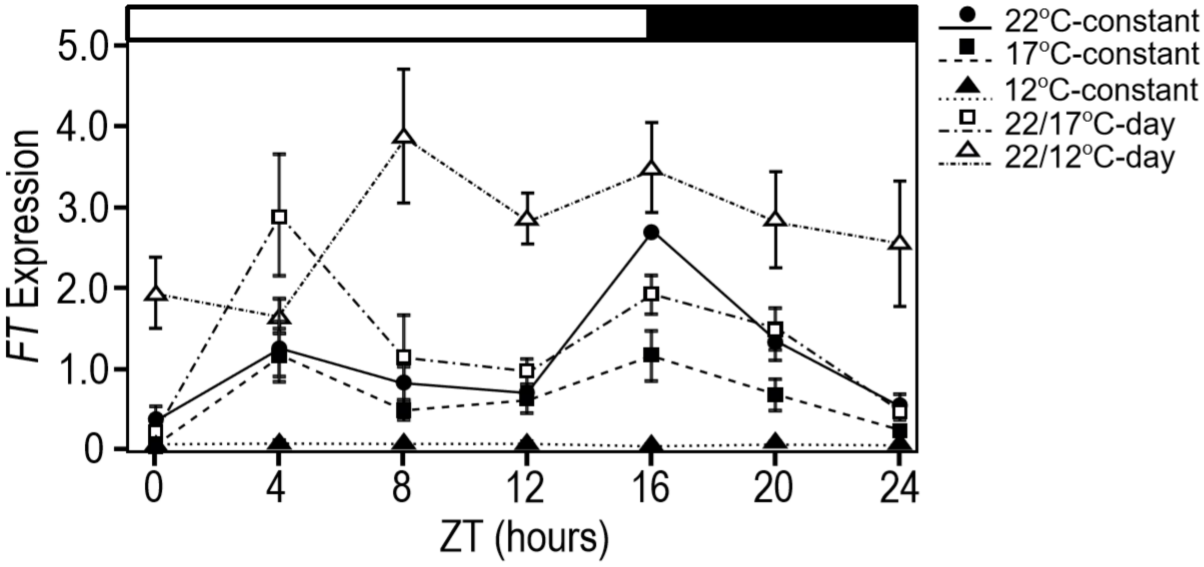

Reference

Kinmonth-Schultz HA, Tong X, Lee J, Song YH, Ito S, Kim S-H, et al. Cool night-time temperatures induce the expression of *CONSTANS* and *FLOWERING LOCUS T* to regulate flowering in *Arabidopsis*. New Phytol. 2016;211:208–24.

S10 Fig. In cool-temperature conditions, in which *FT* expression is suppressed, *FT* accumulation reaches a threshold set in 22˚C-constant conditions only after the influence of leaf age on capacity of different leaves to express *FT* is removed from the model. Plants grown at constant cool (12˚C) temperatures after one week at 22˚C (22 to 12˚C day) accumulate *FT* to a threshold set using 22˚C constant temperatures in long days (thick black line) only after the influence of leaf age on the capacity of leaves to express *FT* is removed from the LTP+GE model variant. Even with this modification, plants grown at cool (12˚C) temperatures from seed (constant) do not attain the threshold.

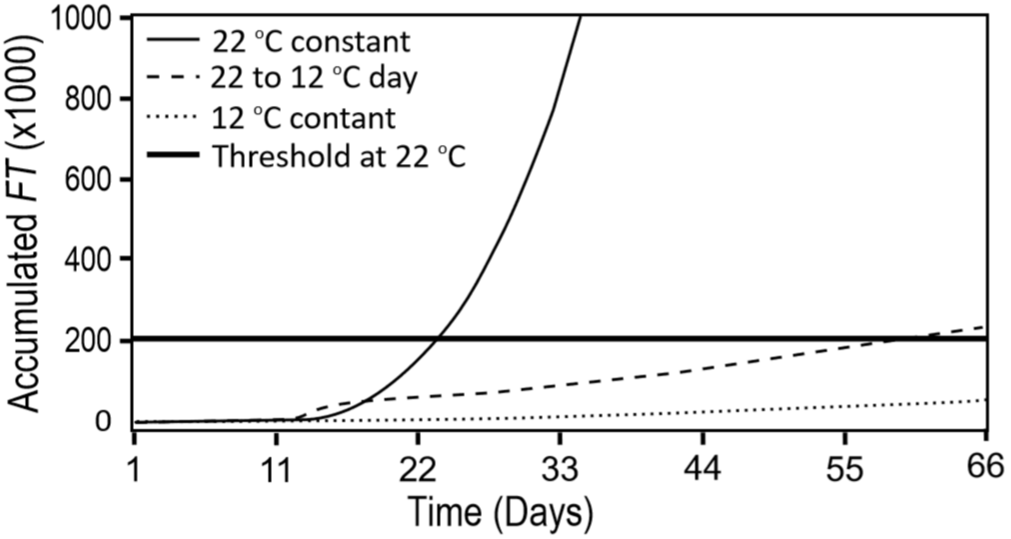

S11 Fig. Growth is slowed and flowering is delayed in plants exposed to 12˚C for two, four, or six days, then returned to warm temperatures (24˚C), relative to control plants grown continuously in warm temperatures. Relative seedling sizes on dawn of day seven, after completion of all cool-temperature treatments (uncropped image).

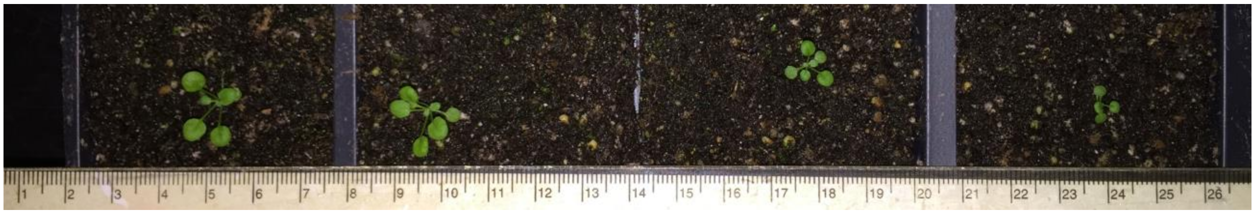

Supplement: 1 — Appendix S1. Equation used in FM-v1.0 to describe FT transcription as a function of CO protein (Chew et al. 2014). Table S1. Coefficients and values for equations used in FM-v1.5. Figure S1. Graphic representation of FM-v1.0. Figure S2. SVP/FLM activity declines over time. Figure S3. Behavior of CO mRNA in response to different temperature regimes. Figure S4. Simulated expression profile of LHY, plotted over time used to increase morning transcriptional sensitivity of FT. Figure S5. Simulated FT expression profile in FM-v1.5 in the svp mutant mimics the pattern but not relative amplitude of that observed. Figure S6. The spatial expression profile of FT changes with leaf age. Figure S7. Behaviour of morphological and physiological parameters in FM-v1.0 and v1.5. Figure S8. Specific leaf area (SLA) declines after growth in cool constant temperatures or in warm-day, cool-night temperature cycles relative to a constant, warm-temperature control. (Pyl et al. 2012). Figure S9. FT expression in plants grown from seed at constant cool temperatures or moved to constant cool temperatures at dawn after 1 week in 22 °C. Figure S10. In cool-temperature conditions, in which FT expression is suppressed, FT accumulation reaches a threshold set in 22 °C-constant conditions only after the influence of leaf age on capacity of different leaves to express FT is removed from the model. Figure S11. Growth is slowed in plants exposed to 12 °C for 2, 4 or 6 days. [file NIHMS1838512-supplement-1.pdf]
